# Gut microbiota: A key player for soluble dietary fiber in regulating inflammatory disease

**DOI:** 10.1016/j.jare.2025.09.030

**Published:** 2025-09-17

**Authors:** Linkai Qu, Ruining Zhang, Ziyu Chu, Jiapei Cai, Yuhang He, Xinyu Zhang, Jiuxi Liu, Xufeng Xie, Yongguo Cao

**Affiliations:** a State Key Laboratory for Diagnosis and Treatment of Severe Zoonotic Infectious Diseases, Key Laboratory for Zoonosis Research of the Ministry of Education, College of Veterinary Medicine, Jilin University, Changchun 130062, China; b Department of Clinical Veterinary Medicine, College of Veterinary Medicine, Jilin University, Changchun 130062, People's Republic of China

**Keywords:** Soluble dietary fiber, Short-chain fatty acids, Gut microbiota, Inflammatory bowel disease, Gut-brain axis, Gut liver axis

## Abstract

Soluble dietary fiber (SDF) plays a significant role in modulating immune responses, particularly in the context of inflammatory diseases. SDF enhances intestinal barrier integrity and regulates immune function by modulating the composition of the gut microbiota and increasing the production of beneficial microbial metabolites, such as short-chain fatty acids (SCFAs), 3-hydroxyoctadecaenoic acid, pentadecanoic acid, and bile acids. Current evidence suggests that SDF holds therapeutic potential in managing chronic inflammatory diseases such as inflammatory bowel disease (IBD), irritable bowel syndrome (IBS), and metabolic dysfunction-associated steatohepatitis (MASH). Despite these promising findings, the mechanisms by which SDF exerts its effects, particularly in distal organs and neuroinflammatory conditions, remain poorly understood. This review explores the mechanistic pathways by which SDF modulates immune regulation and highlights the current challenges and future strategies for optimizing its clinical application in treating inflammatory diseases.

## Highlights

• First summary of soluble fiber's role in gastrointestinal inflammatory diseases.
• First comprehensive review of its role in inflammatory diseases of distal organs.
• Highlights current clinical challenges and potential solutions.

## Graphical abstract

**Figure:**
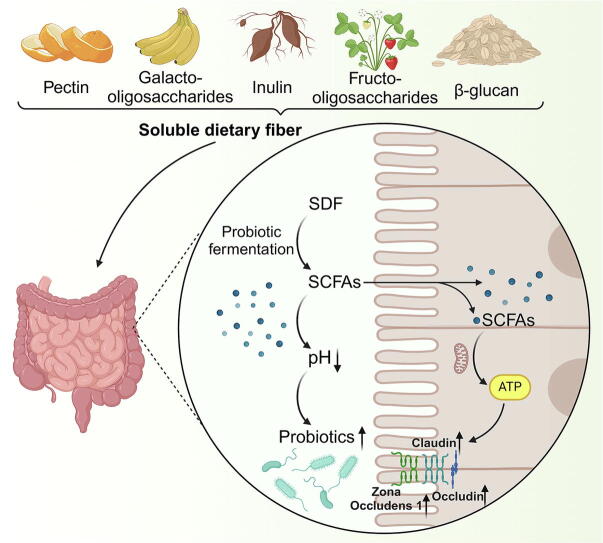

## Introduction

Inflammatory diseases represent a substantial global health challenge, significantly to substantial morbidity and mortality. Despite advances in treatment, current therapeutic strategies exhibit limited ability to provide long-term relief or address the underlying causes of inflammation. The past years, have witnessed a burgeoning interest in the gut microbiota and its pivotal role in modulating immune responses, shedding light on its involvement in both local and systemic inflammation [1,2]. This refined understanding has spurred the development of nutritional immunotherapy, which aims to harness dietary interventions to shape the gut microbiota and enhance immune regulation [3]. Soluble dietary fiber (SDF), a key component of this approach, has attracted significant interest due to its ability to modulate gut microbiota composition and influence inflammatory processes [[4], [5], [6]].

The term “dietary fiber (DF)” was first introduced by the British scientist Eben H. Hipsley in 1953, describing it as the indigestible component of plant cell walls that cannot be absorbed by the human body [7]. During the 1970 s, as research on the health implications of DF gained momentum, scientists began investigating its role in preventing chronic diseases such as obesity, diabetes, and colorectal cancer [[8], [9], [10], [11]]. DF was subsequently into SDF and insoluble dietary fiber (IDF) based on its water solubility in water and its distinct physiological effects on the human body. Despite the growing acceptance of the DF concept within the scientific community, no consensus has been reached on a formal definition existed at that time. It was not until 2009 that the World Health Organization and the Codex Alimentarius established a unified global definition and standard for DF. According to this standard, DF is defined as carbohydrate polymers in food that contain three or more monosaccharide units and are not digested or absorbed in the human small intestine. Besides, they must exhibit at least one beneficial health effect, such as enhancing intestinal motility, increasing fecal bulk, delaying gastric emptying, regulating blood glucose levels, or lowering blood cholesterol levels [12,13]. Furthermore, in 2016, the U.S. Food and Drug Administration refined the 2009 definition, specifying that only fiber components with sufficient scientific evidence supporting their specific health benefits could be classified as DF. This underscored the critical role of scientific evidence in defining dietary fiber. Despite the increasing rigor and scientific basis of DF definitions, discrepancies persist exist in the classification and standards for DF across different countries and organizations. These differences may contribute to inconsistencies in global food labeling and consumer understanding, highlighting the need for continuous updates and harmonization of the DF concept. Moreover, with the shift in modern dietary habits, DF intake has been declining globally [14,15]. The proliferation of highly processed foods and the prevalent fast-food culture have led to reduced consumption of whole grains, vegetables, and fruits, which remain key sources of DF.

An increasing body of epidemiological studies suggests a negative correlation between DF intake and the risk of various diseases, including cardiovascular diseases, metabolic disorders, gastrointestinal conditions, and cancer. A *meta*-analysis that pooled data from nearly 135 million individuals across 185 prospective studies and 58 clinical trials found that high DF intake was inversely associated with all-cause mortality (RR 0.85, 95 % CI 0.79–0.91), coronary heart disease (RR 0.69, 95 % CI 0.60–0.81), stroke (RR 0.80, 95 % CI 0.56–1.14), and cancer mortality (RR 0.87, 95 % CI 0.79–0.95). Moreover, compared to individuals with low fiber intake, those with higher DF intake showed significantly lower incidence rates of coronary heart disease (RR 0.76, 95 % CI 0.69–0.83), stroke (RR 0.78, 95 % CI 0.69–0.88), type 2 diabetes (RR 0.84, 95 % CI 0.78–0.90), and colorectal cancer (RR 0.84, 95 % CI 0.78–0.89) [16,17]. Moreover, a daily DF intake of 25 to 29 g was associated with the lowest disease risk. Given the crucial role of DF in maintaining overall health, most countries recommend a daily DF intake of 25–30 g for adults [18,19]. However, the average daily DF intake among adults worldwide remains below 20 g [20]. Beyond its preventive effects on disease, DF has shown therapeutic potential for gastrointestinal conditions such as inflammatory bowel disease (IBD) and irritable bowel syndrome (IBS), as well as for distal inflammatory diseases like Alzheimer's disease, and metabolic dysfunction-associated steatohepatitis (MASH) [[21], [22], [23], [24]]. Furthermore, due to the unique water-solubility properties of SDF, increasing research attention has focused on the role of SDF in disease prevention and treatment [[25], [26], [27]].

This paper examines the role of gut microbiota in modulating of SDF in inflammatory diseases, including its effects on the gastrointestinal tract and distal organs, as well as its practical applications in clinical settings. Furthermore, our review highlights the challenges encountered by SDF-based intervention strategies and proposes potential solutions. The paper highlights that the therapeutic potential of SDF in inflammatory diseases warrants further investigation and holds significant promise for improving the management and treatment of inflammatory conditions in both the gastrointestinal tract and systemic organs.

## The physicochemical properties of SDF and its impact on gut microbiota

Based on the physicochemical properties and physiological functions of DF, several classification methods have been established, including solubility, viscosity, and fermentability. Recent studies have proposed a more refined classification framework for dietary fibers, emphasizing the need to consider a range of physicochemical properties, including backbone structure (such as degree of polymerization and branching), structural charge (neutral or charged), fermentation rate, and fiber matrix (rigidity, gel formation, or aggregation) [28]. This multidimensional approach allows for a more accurate prediction of the health effects of dietary fibers. For instance, fibers with charged groups, such as pectin, exhibit stronger cation exchange capacities, while fibers with slower fermentation rates, such as certain types of resistant starch, may have more pronounced benefits for distal colon health [29]. However, research on this advanced classification system remains limited, and solubility-based classification continues to be the most commonly used method [30]. SDF is primarily found in fruits, vegetables, legumes, and certain grains, such as pectin, inulin, and β-glucans. Compared to IDF, such as cellulose, hemicellulose, and lignin, SDF exhibits higher solubility in water, forming a viscous gel-like substance [31]. This gel-like property enables SDF to slow down the transit of food through the gastrointestinal tract, thereby assisting in the regulation of blood glucose and cholesterol levels [32]. In addition, SDF undergoes partial or complete fermentation by gut microbiota in the colon, yielding metabolic byproducts such as SCFAs. These SCFAs play crucial roles in modulating host metabolism, enhancing immune function, and maintaining gut health [31,33]. While some studies have demonstrated that certain IDFs also possess physiological functions similar to those of SDF, current research remains focused on SDF [34,35], particularly its role in preventing and treating inflammatory-associated diseases.

**Fig. 1:**
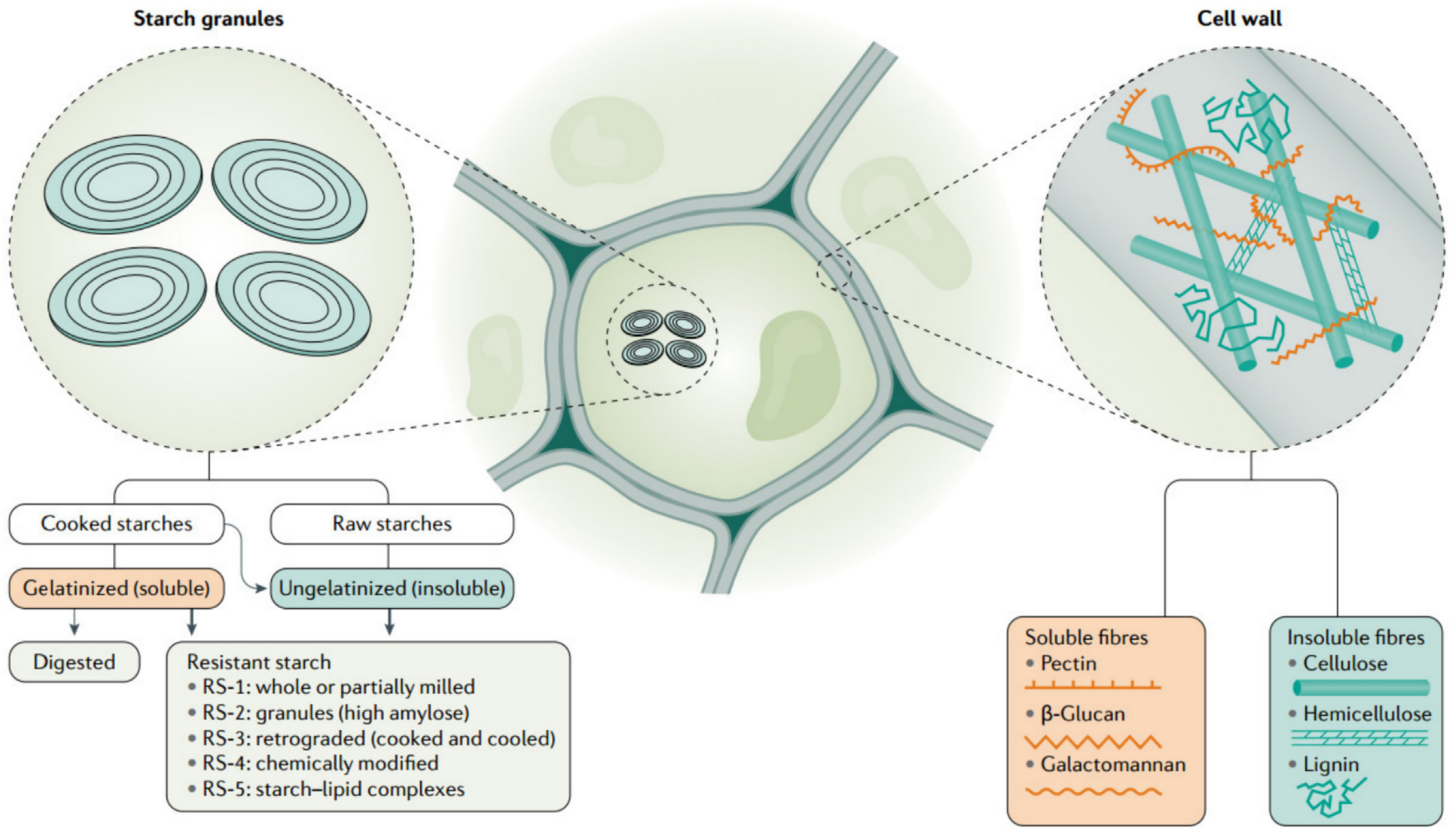
The physicochemical properties of DF and its distribution within plant cells are largely determined by two primary types of polysaccharides: non-starch polysaccharides, which constitute plant cell walls, and resistant starch (RS), which serves as the plant’s energy reserve. The solubility, viscosity, and fermentability of DF are influenced by its chemical structure, interactions with cell wall components, and food processing. These factors are essential in determining fiber’s health benefits, such as gut modulation and metabolic effects. Copyright © 2021, Nature Publishing Group. Replicated with permission from Ref. [42].

Solubility refers to the extent to which DF dissolves in water. Compared to IDF, SDF demonstrates significantly higher hydrophilicity, a property primarily attributed to its unique molecular structure (Fig. 1). SDF consists of various bioactive substances with different molecular configurations, ranging from oligosaccharides, such as fructooligosaccharides (FOS) and galactooligosaccharides (GOS), to high molecular weight polysaccharides, including pectin, β-glucans, inulin, and arabinogalactans. Importantly, in addition to its molecular structure, external factors such as temperature and pH can significantly affect the solubility of SDF [36]. For instance, the solubility of pectin and guar gum increases with the abundance of side chains on their molecular structures [[37], [38], [39]]. Conversely, in the case of β-glucans, an increase in branching can lead to reduced solubility [40]. Additionally, certain SDF are produced through chemical modifications of IDF. A notable example is methylcellulose, where the hydroxyl groups are substituted with methyl groups, thereby enhancing its solubility [41]. Table 1 provides a comprehensive summary of the structure, sources, and physicochemical properties of various SDFs that have been extensively studied and applied in scientific research.

**Table 1:** Structures, sources, and physicochemical properties of different SDF.

SDF	Constituent units	Linkage types	Source	Viscosity	Solubility	Molecular weight range	Fermentability	References
Pectin	Galacturonic acid	α-1, 4-glucoside bond	Apples, citrus fruits	High	High	50–300 kDa	High	[77]
β-Glucan	D-Glucose	β-1,3 and β-1, 4-glucoside bonds	Oats, barley and other grains	Medium-high	Medium-high	50–200 kDa (higher in yeast-derived forms)	High	[78]
Inulin	D-fructose and D-Glucose	β-2, 1-glucoside bond	Chicory, Onions, garlic, bananas and other plants	Low	High	2–10 kDa	High	[79]
Arabinogalactans	L-arabinose and D-Galactose	β-1,3- and β-1, 6-glucoside bonds	Spruce and larch	Low	High	20–100 kDa	High	[80]
Fructooligosaccharides	D-fructose and D-Glucose	β-2, 1-glucoside bond	Chicory, Onions, garlic, bananas and other plants	Low	High	0.5–2 kDa	High	[81]
Guar Gum	D-mannose and D-galactose	D-mannose: Beta-1, 4-glucoside bond；D-galactose: Alpha-1, 6-glucoside bond	*Cyamopsis tetragonoloba*	High	Very high	200–3,000 kDa	Medium-high	[82]
Gum Arabic	It is composed of polysaccharide units such as D-galactose, L-arabinose, L-rhamnose, and D-glucuronic acid	Multiple glucoside linkages	*Acacia senegal*	Low- medium	High	250–600 kDa	High	[83]
Xanthan Gum	D-glucose, D-mannose, and D-glucuronic acid	The main chain is composed of β-1, 4-glucose units, and the side chain is connected in different ways by mannose and glucuronic acid	Fermentation by bacteria (e.g. *Xanthomonas*)	High	High	1,000–2,000 kDa	Low	[84]
Carboxymethyl Cellulose	β-D-glucose	β-1, 4-glucoside bond	Chemically modified natural cellulose	High	High	90–250 kDa (depends on Degree of Substitution)	Low	[85]
Galactooligosaccharides	D-galactose and D-glucose	β-1,4- and β-1, 6-glucoside bonds	Milk and other natural dairy products	Low	High	0.5–3 kDa	High	[86]
Alginate	L-guluronic acid and D-mannuronic acid	Alpha-1, 4-glucoside bond	Brown algae	High	High	50–400 kDa	Low-medium	[87]
Carrageenan	D-galactose and 3, 6-Anhydrogalactose units, usually with a sulfate group	These units are mainly connected by α-1,3- and β-1, 4-glucoside bonds, forming a linear structure	Red alga	High	High	200–500 kDa	High	[88]
Oligofructose	D-fructose	Beta-2, 1-glucoside bond	Garlic, bananas, agave and Jerusalem artichoke	Low	High	1–3 kDa	High	[89]
Trehalose	D-glucose	Alpha-1, 1-glucoside bond	Fungi, algae, invertebrates (such as insects) and some plants	Low	High	342 Da	Low	[90]
Arabinoxylans	D-xylose and L-arabinose	D-xylose: Beta-1, 4-glucoside bond；L-arabinose: Alpha-1,2- or alpha-1, 3-glucoside bond	The ectoderm of grains such as wheat and barley	Medium-high	Medium-high	50–200 kDa	Medium	[91]
Polydextrose	D-glucose	α-1, 4-glucoside bond and other glucoside bonds	Glucose synthesis by polymerization reaction	Low	High	∼3,500 Da	Low-medium	[42]

**Fig. 2:**
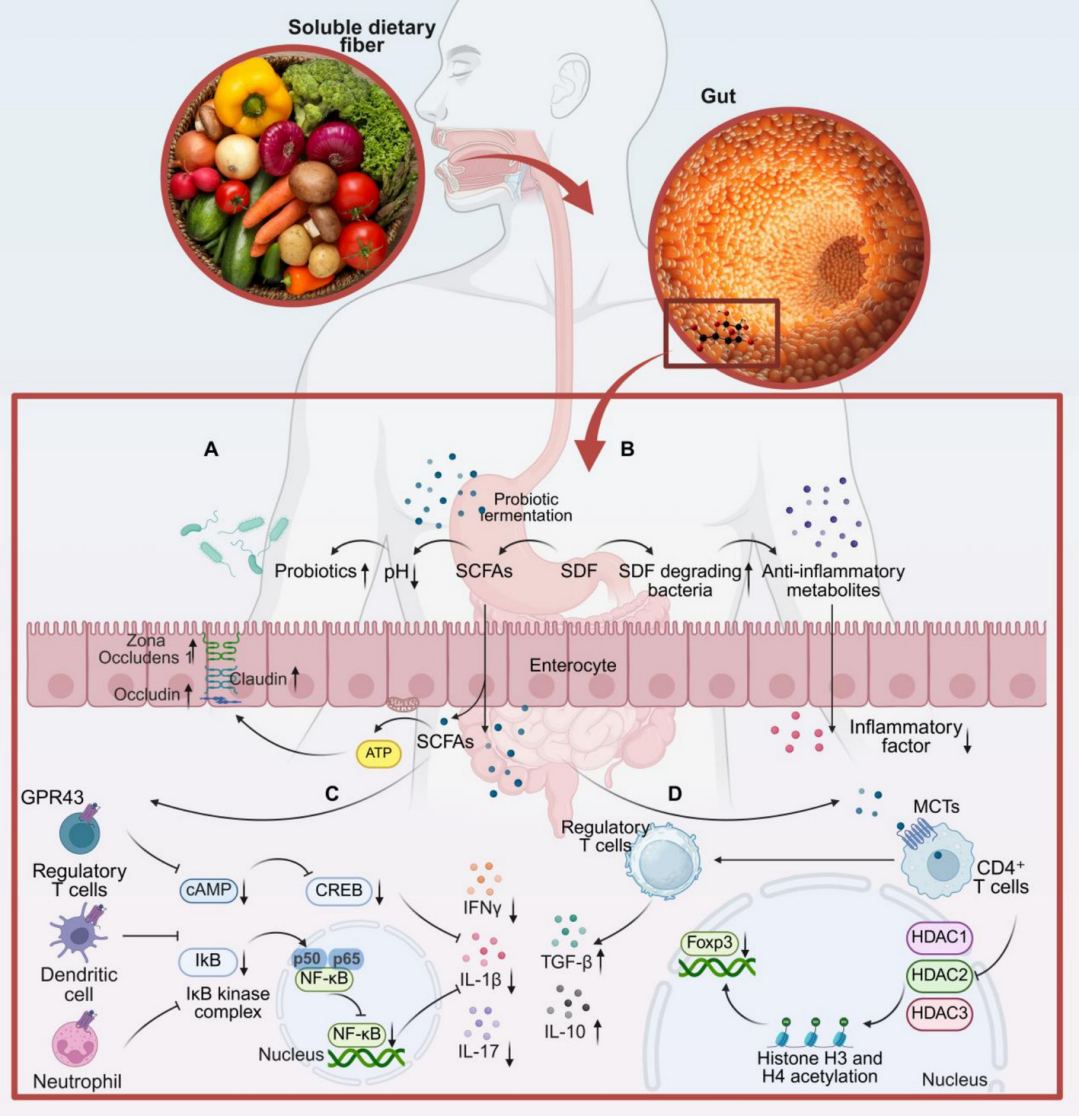
(A) SDF is fermented by gut microbiota to produce SCFAs, which regulate energy metabolism in intestinal epithelial cells and upregulate the expression of tight junction proteins, thereby enhancing gut barrier integrity. Besides, SCFAs lower the intestinal pH, creating an environment favorable for the growth of beneficial bacteria such as Lactobacillus and Bifidobacterium, and consequently reshape the gut microbial composition. (B) Besides SCFA production, the microbial fermentation of SDF generates anti-inflammatory metabolites that suppress the expression of intestinal pro-inflammatory cytokines, alleviating gut inflammation. (C) SCFAs modulate immune responses by activating G protein-coupled receptor (GPCR) 43 and GPR41, expressed on immune cells and intestinal epithelial cells. This activation inhibits the cAMP-CREB and NF-κB signaling pathways, leading to a decrease in the expression of pro-inflammatory cytokines, including TNF-α, IL-6, and IL-1β. (D) In parallel, SCFAs function as inhibitors of histone deacetylases (HDAC1/2/3), enhancing the acetylation of histones H3 and H4. This epigenetic regulation promotes the transcription of the key immunoregulatory gene *Foxp3*, facilitating the differentiation and expansion of regulatory Tregs, and increasing the production of anti-inflammatory cytokines, including IL-10 and TGF-β, thereby contributing to the maintenance of immune homeostasis.

The human gut microbiota refers to the symbiotic community of microorganisms that reside within the human gastrointestinal tract. While current research has primarily focused on bacteria, this ecosystem also encompasses archaea, viruses, fungi, and protists. These microorganisms engage in a multifaceted network of direct and indirect interactions, both with each other and with the host, via physical contact, protein secretion, or metabolic byproduct synthesis, forming a complex and dynamic ecological network [43,44]. This network maintains a balanced microecosystem that is intricately linked to host health and disease. The “balance” or “functional homeostasis” of the gut microbiota is not static but represents a dynamic and functional range indicative of health. Core features of this equilibrium include high microbial diversity, enrichment of beneficial functional taxa (particularly SCFA-producing bacteria), controlled presence of potential pathogens, robust microbial metabolic functions, and resilience against external perturbations [45]. In patients diagnosed with IBD, this functional homeostasis is significantly disrupted. This dysbiosis is characterized by reduced species diversity, depletion of SCFA-producing bacteria, overrepresentation of potential pathogens, and decreased concentrations of SCFAs [46,47]. Such microbial alterations are not only hallmarks of IBD but also key drivers of its pathogenesis. SDFs serve as critical modulators for restoring and maintaining the functional homeostasis of the gut microbiota. Through multiple mechanisms, SDFs influence the dynamic equilibrium of the microbial community and subsequently modulate the abundance of secreted proteins and metabolites in the gut (Fig. 2). First, SDFs serve as primary fermentation substrates for gut microbes, particularly beneficial bacteria such as *Bifidobacterium* and *Lactobacillus*. These bacteria ferment SDF to produce SCFAs, including acetate, propionate, and butyrate [48,49]. The production of SCFAs lowers the luminal pH, creating an acidic environment that inhibits pathogenic bacteria while favoring the growth and colonization of beneficial microbes. Moreover, SCFAs especially butyrate exert direct antimicrobial activity, thereby suppressing the expansion of pathobionts [[50], [51], [52]]. Moreover, SCFAs provide a critical energy source for intestinal epithelial cells, promoting their proliferation and repair. SCFAs also strengthen the intestinal barrier by upregulating the expression of tight junction proteins such as claudins and occludin, thus preserving the integrity of the gut barrier and preventing pathogen invasion [2,53,54]. In terms of immune modulation, SCFAs activate G protein-coupled receptors expressed on immune and epithelial cells, leading to the inhibition of pro-inflammatory signaling pathways such as NF-κB [55,56]. SCFAs also function as histone deacetylase inhibitors, promoting epigenetic reprogramming that favors the differentiation, expansion, and function of regulatory T cells, while simultaneously suppressing pro-inflammatory Th1 and Th17 responses [57]. These effects collectively result in reduced expression of pro-inflammatory cytokines such as TNF-α, IL-6, IL-1β, IL-17, and IFN-γ, and increased production of the anti-inflammatory cytokine IL-10. This immunological rebalancing is critical for alleviating the exaggerated inflammatory response and tissue injury characteristic of IBD. In addition to these pathways, immunoglobulin A (IgA) responses play a pivotal role in maintaining mucosal immunity. SCFAs have been shown to enhance the production of secretory IgA, which is crucial for defending against pathogens in the gut while also modulating the gut microbiota [58]. Direct research evidence has shown that soluble dietary fiber-garlic polysaccharide increases IgA levels in a dose-dependent manner [59]. This IgA-mediated immune response is integral to the protection of the intestinal barrier and the regulation of host-microbiota interactions. In addition to SCFAs, microbial-derived metabolites, including tryptophan-derived indole compounds and D-amino acid metabolism, also play significant roles in regulating immune responses and maintaining gut homeostasis. Tryptophan-derived indoles, produced by gut microbiota, influence immune modulation by activating the aryl hydrocarbon receptor (AhR) pathways [60,61]. Activation of AhR by these indoles has been shown to regulate the differentiation of T regulatory cells (Tregs) and enhance mucosal immunity by promoting the expression of anti-inflammatory cytokines, such as IL-10 [62]. This pathway is crucial for maintaining intestinal homeostasis and preventing excessive inflammatory responses that could lead to autoimmune or inflammatory diseases. Furthermore, D-amino acids, produced through microbial fermentation of amino acids, modulate the gut microbiota composition by influencing bacterial peptidoglycan metabolism and regulating microbial diversity [63,64]. These metabolites have been found to affect biofilm formation and pathogen colonization, indirectly affecting immune responses by enhancing the production of antimicrobial peptides and regulating the activity of intestinal epithelial cells [64,65]. The combined effects of these microbial metabolites, along with SCFA production, help to maintain a balanced immune response, supporting the protective role of the gut microbiota against inflammatory diseases. Variations in molecular structure among different types of SDF exhibit distinct fermentation properties, selectively promoting the growth of specific bacterial populations. For instance, FOS and GOS are classified as prebiotics, which selectively stimulate the growth of beneficial bacteria such as *Bifidobacteria* and *Bacteroide* [66,67]. Inulin, another type of SDF, significantly enhances gut microbial diversity and stability by increasing the abundance of *Bifidobacterium* [68].

In addition to SCFAs, the fermentation of SDFs by gut microbiota also yields other byproducts such as lactic acid, hydrogen, and carbon dioxide, which can modulate gut motility and indirectly influence microbial distribution [69]. Beyond these effects, recent studies have highlighted the critical role of oxygen concentration along the gastrointestinal tract in shaping microbial composition. In a healthy gut, oxygen levels progressively decrease from the duodenum to the colon, establishing a longitudinal oxygen gradient maintained by host physiological mechanisms rather than microbial consumption [70,71]. In the small intestine, relatively higher oxygen and nitrate availability support the growth of facultative anaerobes through respiration, whereas in the colon, host-mediated epithelial hypoxia (<1% O_2_) limits oxygen diffusion into the lumen, thereby promoting the dominance of obligate anaerobic fermenters such as *Clostridia* and *Bacteroidia* [[72], [73], [74]]. Disruption of this physiological hypoxia, as observed during inflammation or under high-fat, low-fiber diets, leads to increased luminal oxygen and nitrate levels, favoring the overgrowth of facultatively anaerobic pathogens, including *Enterobacteriaceae*, and contributing to dysbiosis [75]. Thus, dietary fiber not only provides fermentation substrates but also indirectly contributes to the maintenance of luminal anaerobiosis, thereby supporting a homeostatic, health-associated microbiota and concurrently suppressing oxygen-fueled pathogen expansion. Furthermore, as the complexity of SDFs s increases, a proportional increase is observed in the number of microbial carbohydrate-active genes that exhibit substrate specificity [76].

Based on the aforementioned mechanisms, SDFs exerts their influence on the gut microbiota through fermentation processes, the production of metabolic byproducts, and their direct and indirect effects on the host. These interactions ultimately impact the host's health, particularly in areas such as gut health, immune function, and metabolic regulation. Therefore, increasing SDF intake in the diet is a crucial strategy for improving the gut microbiota and enhancing overall health.

## The role of SDF in gastrointestinal inflammatory diseases

**Fig. 3:**
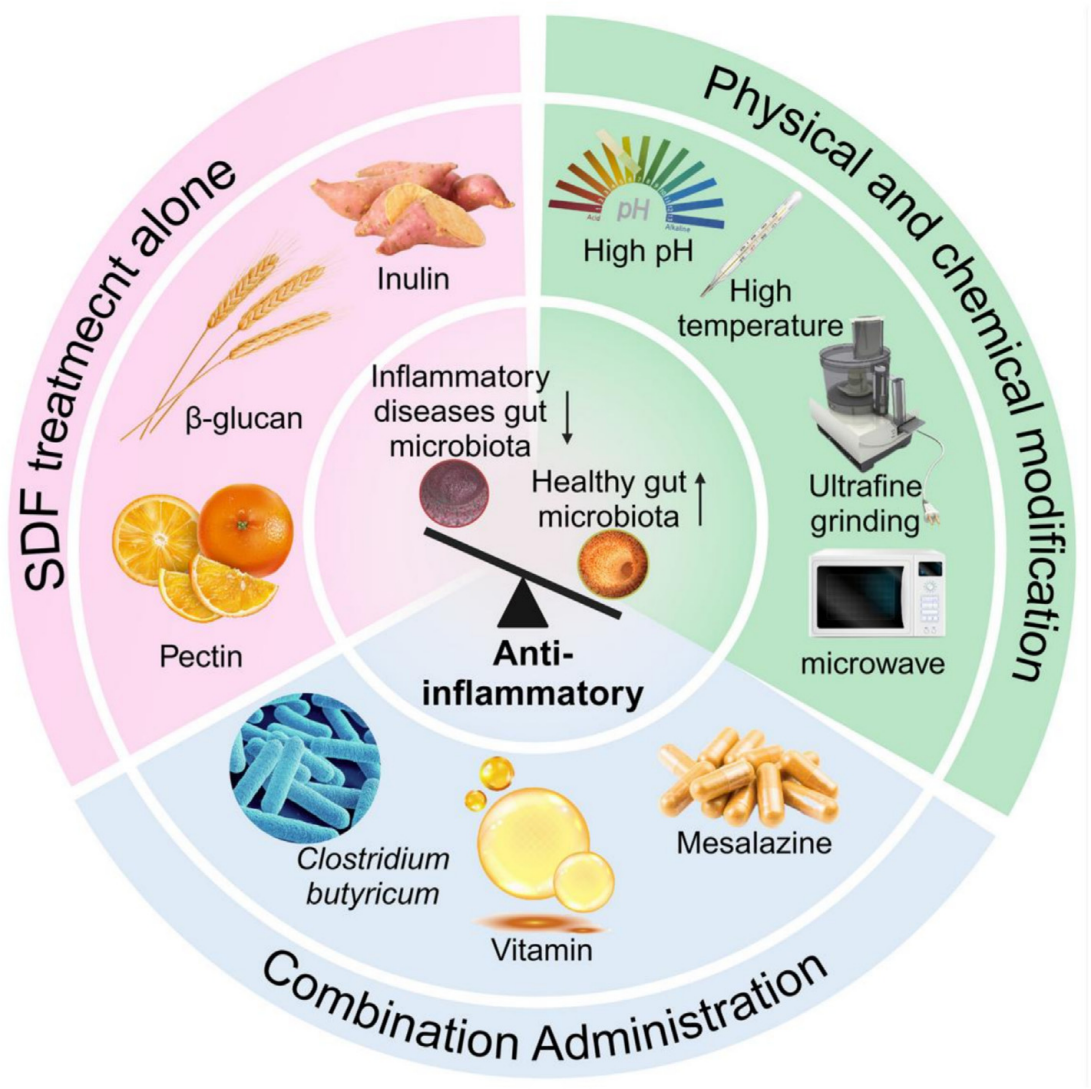
Treatment methods and therapeutic strategies of different SDFs for inflammatory diseases. Therapeutic strategies comprise three primary categories: (1) SDF monotherapy exerting protective effects against inflammatory diseases; (2) Enhancement of therapeutic efficacy for inflammatory diseases through physicochemical modification of SDFs lacking or exhibiting weak intrinsic protective effects; (3) Combination therapy integrating SDFs with established therapeutic approaches to improve treatment outcomes for inflammatory diseases.

Regulation of SDF intake remains a commonly used approach in the clinical treatment of various gastrointestinal diseases (Fig. 3) [[92], [93], [94]].

### Fiber-specific mechanisms and disease-relevant targets

SDFs are polysaccharides characterized by diverse structural and physicochemical properties (Table 1), which significantly influence their fermentability, microbiota specificity, metabolite profiles, and downstream immunological effects. Increasing evidence suggests that different types of SDFs exert disease-specific therapeutic effects via distinct mechanistic pathways.

Pectin is a highly fermentable plant-derived polysaccharide rich in galacturonic acid that effectively promotes the growth of *Bacteroides* [95,96]. Its fermentation leads to the generation of SCFAs, including acetate and butyrate, which can enhance intestinal epithelial integrity via upregulation of tight junction proteins and mucin secretion [97,98]. Butyrate further activates GPR41, GPR43, and PPARγ, thereby exerting immunomodulatory effects by reducing pro-inflammatory cytokines (e.g., IL-6, TNF-α) and alleviating colitis symptoms in murine models [99]. Given the robust evidence of butyrate's efficacy in colitis models, pectin fermentation-derived SCFAs, particularly butyrate, are increasingly recognized as key mediators in ameliorating intestinal inflammation [100].

Inulin, a fructan-type SDF composed of β(2 → 1) fructosyl units, selectively stimulates *Bifidobacteria* resultsing in increased propionate and butyrate production [[101], [102], [103]]. Propionate acts on GPR41 and inhibits histone deacetylase, modulating gene expression involved in Treg differentiation and mucosal immunity [104,105]. In metabolic inflammation models—including obesity, type 2 diabetes, and MASH inulin has demonstrated the potential to reduce adipose tissue inflammation, improve insulin sensitivity, and decrease intestinal permeability through restoration of tight junction proteins such as claudin-1 and occludin [103,106,107]. Besides, in IBD models, inulin supplementation has been associated with improved epithelial barrier function and reduced mucosal inflammation [108]. However, its strong fermentability may provoke gastrointestinal side effects in sensitive individuals, such as those with IBS.

β-glucan, a glucose polymer characterized by β-1,3 and β-1, 4-glycosidic bonds and derived from sources such as oats, barley, and fungi, exhibits both prebiotic and immunoregulatory properties. Unlike pectin and inulin, β-glucan interacts directly with Dectin-1 receptors on dendritic cells and macrophages, promoting anti-inflammatory signaling pathways, including the induction of IL-10 and the suppression of NF-κB activation [[109], [110], [111]]. It also enhances phagocytic function and mucosal defense, demonstrating efficacy not only in colitis models but also in neuroinflammation and liver injury via the gut-liver and gut-brain axes, respectively [[112], [113], [114], [115]]. Importantly, β-glucan has shown synergistic effects with SCFAs, leading to amplification of epithelial barrier repair mechanisms [116,117].

In addition to pectin, inulin, and β-glucan, other SDFs, such as FOS and GOS, have demonstrated anti-inflammatory potential in various inflammatory disease models. However, current evidence regarding these fibers remains limited. Further research is needed to elucidate their underlying mechanisms and evaluate their therapeutic efficacy in specific disease contexts.

### Irritable bowel syndrome

**Fig. 4:**
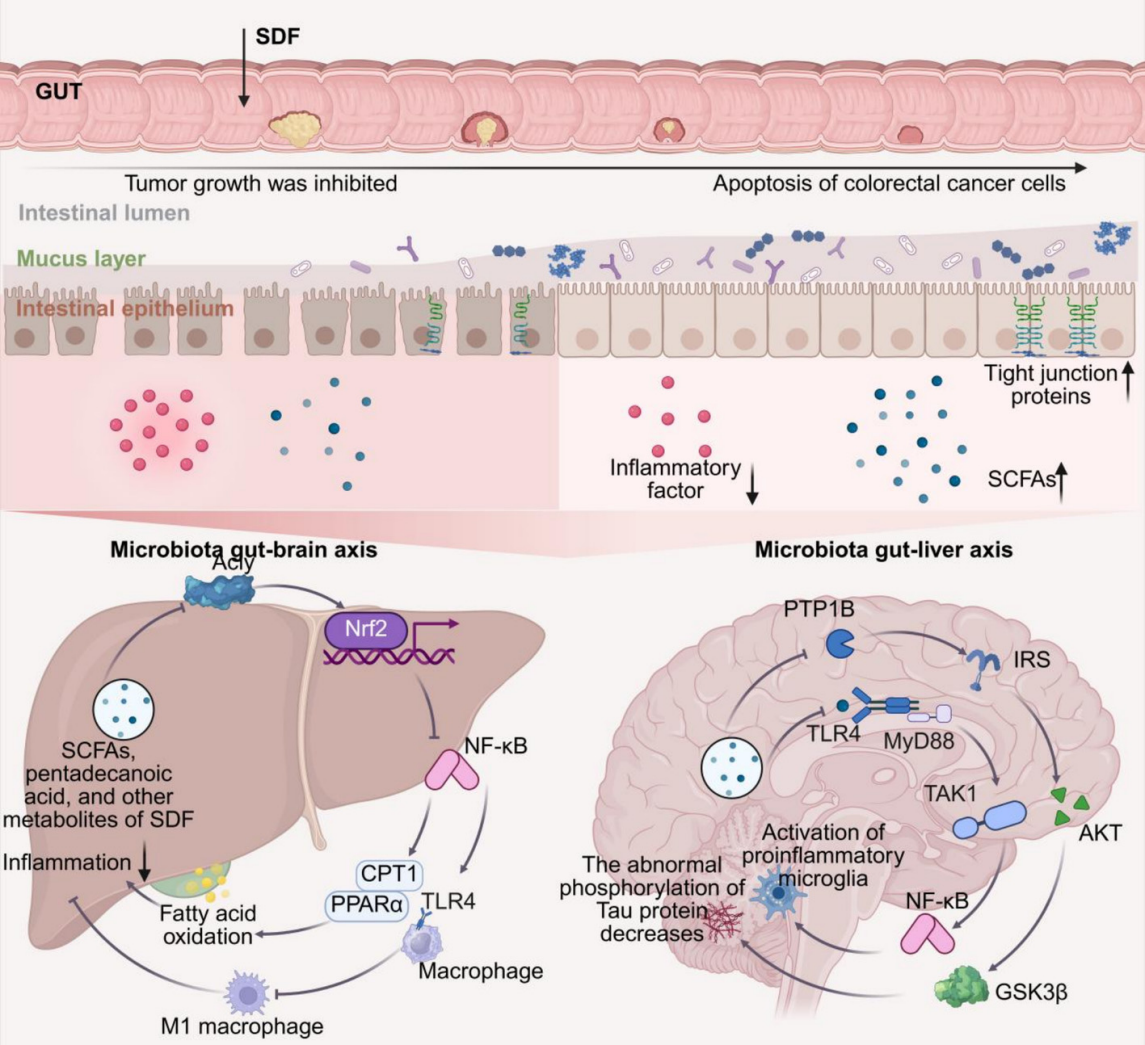
Mechanistic diagram of the therapeutic effects of SDF on different inflammatory diseases. Various types of SDF regulate gut microbiota through microbial fermentation, producing beneficial metabolites that improve intestinal permeability and reduce inflammation. Besides, SDF acts through the gut-liver and gut-brain axes to decrease liver and neuroinflammation, thereby ameliorating pathological features.

IBS is a functional gastrointestinal disorder characterized by abdominal pain associated with constipation, diarrhea, or a combination of both [118]. Epidemiological studies have shown that the global prevalence of IBS in women is 12 % (95 % CI, 9.3 %–15 %), which is slightly higher than in men, with a prevalence of 8.6 % (95 % CI, 6.3 %–11.2 %) (odds ratio 1.46; 95 % CI 1.33–1.59). Additionally, research indicates that women are more likely than men to seek healthcare services for IBS and other functional gastrointestinal disorders [119]. IBS significantly affects patients' quality of life and work productivity. In the U.S., IBS prevalence ranges from 7 % to 16 %, with the associated direct annual costs reaching up to $950 million [[120], [121], [122]]. While IBS is not strictly classified as an inflammatory disease, IBS patients often exhibit inflammation-related symptoms, such as abdominal pain and gastrointestinal discomfort [123,124]. Fiber intake guidelines for IBS patients vary across countries. However, a common recommendation involves reducing the intake of IDF while increasing SDF consumption [92,94,125,126]. To date, many *meta*-analyses and systematic reviews have concluded that certain types of DF may help alleviate IBS symptoms and improve stool frequency and consistency. While the variability in response remains a challenge, the evidence consistently points to the beneficial role of SDF [127,128]. A *meta*-analysis of 14 randomized controlled trials (RCTs) involving 906 patients evaluated the role of fiber in IBS and found that SDF exhibited a therapeutic effect on IBS compared to IDF (RR = 0.83; 95 % CI 0.73–0.94) [129]. This finding was endorsed by the American College of Gastroenterology Guidelines, which in 2021 incorporated the use of SDF (but not IDF) into its management recommendations for IBS [130]. A study by Bijkerk CJ et al. also found that supplementing IDF could worsen IBS symptoms [131]. However, another *meta*-analysis of 11 RCTs indicated that, despite an increase in *Bifidobacterium* abundance after SDF supplementation, there was no significant improvement in the severity of abdominal pain, bloating, flatulence, or overall IBS scores. Notably, lower doses of SDF, especially non-inulin-type fructans like GOS and partially hydrolyzed guar gum, have been shown to reduce bloating [132]. Furthermore, some studies suggest that the viscous properties of SDF result in a slower fermentation rate in the gastrointestinal tract, potentially potential therapeutic benefits for IBS [133,134] (Fig. 4). While these findings suggest a promising therapeutic role for SDF in IBS, inconsistencies in SDF dosing and patient populations, as well as a lack of large-scale clinical studies, necessitate further research to fully establish the efficacy of SDF in treating IBS. In addition to IBS, SDF plays a significant role in managing another common gastrointestinal disorder: IBD.

### Inflammatory bowel disease

IBD, encompassing Crohn’s disease (CD) and ulcerative colitis (UC), is a persistent and recurrent inflammatory condition affecting the gastrointestinal tract. Its etiology involves a complex interplay of host genetics, microbial composition, and environmental factors, all of which are essential for the onset of IBD. This complexity makes the pathogenesis of IBD difficult to fully elucidate, and the underlying mechanisms are not yet fully understood [22]. Historically, since the onset of industrialization, the incidence of IBD has steadily increased, suggesting that its rise is unlikely to be solely attributable to genetic factors or natural selection [135]. Instead, rapid changes in human lifestyle, diet, and environmental exposures are believed to contribute to the rising prevalence of these diseases. The composition and function of the gut microbiota, being highly sensitive to dietary and environmental factors, play a crucial role in the development of immune imbalance in genetically predisposed individuals, ultimately promoting the onset of IBD [22]. The prevalence of IBD in North America and Europe currently exceeds 0.3 % [136]. Moreover, epidemiological studies predict that by 2030, the prevalence of IBD could reach as high as 1 % in some parts of the Western world [137]. Given this alarming trend, the need for effective strategies to manage and treat IBD has become increasingly urgent.

DF is considered a key determinant in addressing intestinal inflammation [92,94,138]; however there is ongoing debate regarding its clinical application in IBD. Current research largely concurs that DF, particularly SDF, is fermented by gut microbiota to produce metabolites such as propionate and butyrate. These metabolites modulate inflammatory responses by upregulating anti-inflammatory cytokines like IL-10 and downregulating pro-inflammatory cytokines such as IFN-γ, IL-1β, and TNF-α, thereby alleviating intestinal inflammation and regulating gut immunity [[138], [139], [140], [141], [142], [143], [144]]. Studies have found that fecal concentrations of SCFAs are significantly lower in IBD patients compared to healthy individuals, suggesting that reduced fermentation capacity in IBD patients may result from gut dysbiosis, leading to decreased SCFA production [145,146]. Moreover, patients with UC typically exhibit lower abundance of beneficial bacteria such as *Bifidobacterium*, while SDF supplementation can help restore these microbial populations [48,49,147]. Regarding IBD risk, a prospective study involving an average follow-up of 12.1 years identified 543 cases of CD and 939 cases of UC. The authors reported a negative correlation between DF intake and IBD risk (HR 0.74, 95 % CI 0.58–0.93, p = 0.011) and CD risk (HR 0.48, 95 % CI 0.32–0.72, p < 0.001), but no significant association with UC risk (HR 0.92, 95 % CI 0.69–1.24, p = 0.595) [148]. Conversely, another prospective study with a 26-year follow-up of 170,776 women yielded different results. Among the 269CD and 338 UC cases identified, women in the highest quintile of DF intake (median 24.3 g/day) demonstrated a 40 % reduced risk of UC (hazard ratio for CD, 0.59, 95 % CI 0.39–0.90), but no significant association with CD [149]. Notably, this risk reduction was linked to DF intake from fruits, while DF from grains, whole grains, or legumes did not show similar protective effects [149]. This distinction suggests that SDF, which is abundant in fruits, may play a more critical role in IBD compared to IDF, found in higher concentrations in grains, whole grains, and legumes [131,132]. In clinical treatments for IBD, only a few studies have reported the effects of DF on symptom alleviation and disease management. In UC management, a large RCT found that psyllium (SDF), mesalamine, and a combination of psyllium and mesalamine all provided remission over a 12-month period [150]. Furthermore, another study demonstrated that patients treated with mesalamine alone had a higher failure rate compared to those receiving a combination of psyllium and mesalamine [151]. Two additional studies also supported the beneficial role of DF, particularly SDF, in UC remission [151,152]. In contrast, studies examining the role of DF in CD have shown mixed results. One study found no significant difference in disease control between high-fiber and low-fiber groups over a 24-month period [153]. Another study reported that patients on a high-fiber diet (33.4 ± 1.8 g per day, with 2.9 ± 0.3 g from raw fruits and vegetables) experienced a higher treatment failure rate and shorter remission time compared to those on a low-fiber diet, suggesting that high fiber intake may not be beneficial for UC [154]. However, this study overlooked the critical aspect of SDF intake, as the fiber consumed was predominantly IDF rather than SDF [154]. FOS upregulates the production of 3-hydroxyoctadecaenoic acid in the gut, which acts as an agonist of peroxisome proliferator-activated receptor γ (PPARγ) to exert anti-inflammatory effects [155]. In addition to its effects on colitis, co-administration of *Lactiplantibacillus plantarum* L47 and inulin has been shown to possess therapeutic potential against ileitis. This therapeutic effect is mediated by the upregulation of α-linolenic acid and 12,13-epoxyoctadecenoic acid levels, which help mitigate ileal inflammation induced by enterotoxigenic *Escherichia coli* in piglets [156].

In summary, while the mechanisms by which DF affects IBD are not yet fully understood, the available evidence suggests that, after careful consideration of other potential risks, IBD patients may benefit more from increasing their SDF intake rather than IDF. This approach appears to offer superior therapeutic potential for the disease management.

### Colorectal cancer

Colorectal cancer is a type of malignant tumor typically caused by a combination of genetic factors, dietary habits, IBD, or environmental influences. This category includes cancers of the colon, rectum, and small intestine [157]. Colorectal cancer is one of the three most common cancers worldwide [158]. Epidemiological data from 2023 indicate that approximately 153,020 people in the United States were diagnosed with colorectal cancer, with an estimated 52,550 deaths expected from the disease [159]. A *meta*-analysis involving 15,740 adults revealed a negative correlation between SDF intake and colorectal cancer mortality[160]. A similar conclusion was drawn by Arayici et al., who reported that SDF significantly reduced the risk of colorectal cancer (ES 0.78, 95 % CI 0.66–0.92) [161]. In terms of specific mechanisms, research has shown that pectin enhances the efficacy of anti-PD-1 antibodies in colorectal cancer by modulating T-cell infiltration in the tumor microenvironment, a function potentially mediated by the production of butyrate, a metabolite of pectin fermentation [162]. The overexpression of galectin-3 is closely linked to colorectal cancer progression, and pectin extracted from papaya has been found to bind and inhibit galectin-3, reducing the proliferation of colorectal cancer cells. Interestingly, this effect was shown to be dependent on the ripeness of the papaya [163]. Moreover, foxtail millet SDF has been reported to exhibit a beneficial effect on colorectal cancer. A 10-week study involving the administration of 200 mg/kg foxtail millet SDF demonstrated apoptosis-promoting effects in HT-29 colon cancer cells by regulating the mitochondrial apoptosis pathway, alleviating symptoms in mice with colorectal cancer [164]. Oat β-glucan, a component of SDF, exhibits broad biological activities, including anti-inflammatory and anti-tumor properties. Studies have shown that 3 % oat β-glucan stimulates autophagy and the extrinsic apoptosis pathway in colorectal cancer cells, while also enhancing the intrinsic apoptosis pathway[165]. Resistant starch, widely recognized for its ability to regulate gut function, blood glucose, and lipid metabolism, has also been reported to play a role in colorectal cancer prevention. These effects are mediated by inhibiting the expression of carcinogenesis-related proteins such as heat shock protein 25, protein kinase C-delta, and gastrointestinal glutathione peroxidase in colon epithelial cells. Moreover, resistant starch increases oxidative and endoplasmic reticulum stress, activating transcription factor 4 and secretase beta expression, which enhances apoptosis in colon tumor cells [166]. Beyond the effects of individual SDF components, modified SDF have also shown promise in cancer prevention. For example, high-pH and high-temperature-modified citrus pectin inhibits galectin-3 expression and suppresses sugar-mediated tumor growth, angiogenesis, and metastasis *in vivo*, with a significant dose-dependent protective effect [167]. Chen et al. found that heat-treated citrus pectin triggers mitochondrial oxidative stress by inhibiting the activity of succinate-ubiquinone reductase in mitochondrial complex II, thereby promoting colorectal cancer cell death [168]. However, some studies have yielded contradictory findings. For instance, Yang et al. reported that inulin and guar gum increased fecal butyrate and serum bile acid levels, lowered inosine levels, and accelerated tumorigenesis in colorectal cancer mouse models. They also found a positive correlation between inulin dosage and tumor formation [169]. Similarly, Okazaki et al. found that guar gum promoted colorectal tumor development [170]. This discrepancy may be attributed to the excessive intake of a single type of SDF, such as inulin, which may disrupt the balance of gut fermentation and lead to the overproduction of specific microbial metabolites, such as secondary bile acids or lactate, which could contribute to epithelial damage or promote oncogenesis. Besides, the absence of IDF in the intervention diet may exacerbate the negative effects of SDF. Insoluble fibers can help modulate fermentation kinetics, buffer excessive SCFA or bile acid accumulation, and support healthy gut transit time. Therefore, a balanced intake combining both soluble and insoluble fibers may be essential to harness the benefits of SDF while minimizing risks. Taken together, these findings underscore the importance of proper fiber selection, dose control, and dietary context in clinical and experimental applications of SDF.

Overall, while the precise mechanisms by which SDF influences colorectal cancer remain to be fully understood, and some studies have reported contrasting findings, there is growing evidence suggesting that, upon careful consideration of other risks, controlled SDF supplementation may offer benefits to colorectal cancer patients. Besides, its established role in preventing and treating gastrointestinal inflammatory diseases, SDF may also have therapeutic and preventive effects on distal inflammatory conditions.

## The role of SDF in alleviating inflammation in extra-intestinal organs

SDF regulates gut microbiota and is fermented by these microorganisms to produce metabolites such as SCFAs. Alterations in the gut microbiota and its metabolic products have been identified as risk factors for distal inflammatory diseases, such as neuroinflammation and MASH. Increasing evidence suggests that SDF-mediated modulation of the gut microbiota exerts significant therapeutic effects in extra-intestinal organs, including the brain and the liver.

### Neuroinflammation in the brain

Neuroinflammation is defined as an inflammatory response in the central nervous system (CNS) mediated by the activation of neurons, microglia, astrocytes, and other immune cells [171]. This chronic low-grade inflammation is closely associated with the pathogenesis of several neurodegenerative diseases, including Alzheimer’s disease, Parkinson’s disease, and multiple sclerosis. Among CNS-resident immune cells, microglia and astrocytes play central roles in initiating and propagating neuroinflammation. As the innate immune sentinels of the brain, microglia are distributed throughout the CNS and are critical for maintaining immune homeostasis and supporting synaptic plasticity [172].

SCFAs, primarily produced through anaerobic fermentation of SDFs and indigestible carbohydrates by gut microbiota in the colon [173], are essential for microglial maturation and immune modulation [174,175]. Notably, butyrate has been demonstrated to reduce the expression of pro-inflammatory cytokines in microglia from aged mice. In a dietary intervention study involving mice fed with 5 % inulin exhibited improved gut microbiota composition, increased levels of butyrate, acetate, and total SCFAs, along with a significant downregulation of neuroinflammatory markers [176]. Consistent with these findings, Vailati-Riboni et al. reported that 5 % inulin supplementation restored microglial gene expression and normalized TNF-α secretion through enhanced SCFA production. [177]. Mechanistically, X.-W. Li et al. found that inulin attenuates neuroinflammation induced by 2-ethylhexyl diphenyl phosphate through inhibition of the TLR4/NF-κB signaling pathway [[178], [179], [180], [181]]. Moreover, neuroinflammation associated with postpartum depression was alleviated by a 0.037 g/kcal inulin diet, which elevated SCFA levels, improved depressive-like behaviors, and restored endocrine balance [182]. Mechanistic investigations by Wang et al. (2023) revealed that inulin-induced SCFA production contributed to the maintenance of blood–brain barrier (BBB) integrity and attenuated CNS inflammation via the TLR4/MyD88/NF-κB signaling pathway. The study further identified a positive correlation between SCFA levels and behavioral improvement and BBB integrity, and a negative correlation with neuroinflammatory responses [183]. In addition to its therapeutic potential, inulin has been explored as an adjuvant in inactivated encephalitis vaccines, providing heterologous protection against viral encephalitis [[184], [185], [186]]. β-Glucan, another well-characterized SDF, has demonstrated notable efficacy in mitigating neuroinflammation across multiple models. Long-term supplementation with 7 % β-glucan effectively suppressed high-fat diet–induced microglial activation, synaptic engulfment, and pro-inflammatory cytokine overexpression, while enhancing synaptic remodeling through activation of the PTP1B–IRS–AKT–GSK3β–Tau pathway [113]. At a dose of 50 mg/kg/day, β-glucan significantly reduced microglial and astrocyte hyperactivation and ameliorated neuroinflammation [187]. *In vitro* assays further supported its immunomodulatory function, where 10 μg/mL of β-glucan co-cultured with microglia enhanced immune tolerance [188]. Sources of β-glucan exhibit conserved biological effects. For example, *Lentinula edodes*–derived β-glucan (1.5 mg/day) reversed high-fat diet–induced synaptic structural abnormalities, reduced neuroinflammation, restored brain-derived neurotrophic factor (BDNF) levels, and decreased colonic pro-inflammatory macrophage infiltration [189]. Similarly, yeast-derived β-glucan (100 mg/kg) modulated the gut microbial composition and metabolite profile, reducing cortical and hippocampal inflammation and microglial activation via the gut–brain axis [190]. It also enhanced SCFA production, improving brain insulin sensitivity and reducing neuroinflammatory responses [191]. These findings have been translated into clinical research. A randomized trial demonstrated that co-administration of 250 mg yeast β-glucan with multivitamins improved disease markers in patients with cognitive impairments [192]. Although inulin and β-glucan are the most extensively studied SDFs, mechanistic insights into their roles in neuroinflammation remain limited. In addition, other SDFs, such as pectin, FOS, and GOS, have shown potential in alleviating neuroinflammation. However, most evidence remains preliminary, necessitating further mechanistic and clinical studies are needed to validate their therapeutic potential [[193], [194], [195], [196], [197], [198], [199], [200], [201], [202], [203]].

### Non-viral inflammation of the liver

Non-viral hepatitis represents a distinct category of liver inflammation, differentiating itself from viral hepatitis by its etiology, which includes metabolic dysfunction, drug toxicity, autoimmune reactions, or environmental influences. This category includes diseases such as metabolic associated fatty liver disease (MAFLD), drug-induced hepatitis, alcoholic hepatitis, and autoimmune hepatitis [204]. Among these, MAFLD is the most prevalent and frequently reported in the context of SDF treatment. MAFLD is strongly associated with obesity, type 2 diabetes, and metabolic syndrome [205]. The early stages of MAFLD involves fat accumulation in the liver, which can progress to MASH as hepatocellular damage and inflammation intensify [206]. Studies have shown that SDF, such as inulin, is more effective than IDF, such as cellulose, in inhibiting the progression of MASH in mice, alleviating necrotic inflammation and liver fibrosis [207]. Mechanistically, was found to be metabolized by *Parabacteroides distasonis* into pentadecanoic acid, which exerts anti-inflammatory effects by inhibiting NF-κB activation induced by MASH and suppressing the expression of pro-inflammatory cytokines such as CCL2, CXCL2, and CXCL10 [207]. Moreover, *Dendrocalamus brandisii* (Munro) Kurz shoot SDF (DS-SDF) has been reported to significantly improve MASH in mice by reducing liver inflammation and increasing gut microbiota diversity, beneficial bacterial abundance, and SCFAs production after eight weeks of feeding [208]. However, the specific mechanisms by which DS-SDF improves MASH were not explored in this study. Sodium alginate, an SDF extracted from brown algae, has demonstrated beneficial effects in MASH by improving hepatic steatosis and mitigating liver inflammation, likely through the suppression of F4/80^+^ macrophage infiltration and downregulation of Tnf-α mRNA expression [209]. Tomato pectin has also been found to modulate hepatic bile acid metabolism by altering fecal bile acid composition, resulting in significant changes in functional conjugated bile acids. This, in turn, suppresses the ileal farnesoid X receptor (FXR)/fibroblast growth factor 15 (FGF15) signaling pathway. FXR serves as a central regulator of bile acid metabolism that influences lipid homeostasis and glucose balance. Its activation has been associated with reduced hepatic steatosis, improved insulin sensitivity, and inhibition of inflammation and fibrosis, thereby exerting anti-inflammatory effects [210]. Beyond the effects of individual SDFs, combinations of probiotics and SDFs have also been investigated for treating MASH. For example, a mixture of *Clostridium butyricum* and various SDFs (GOS, stachyose, and mannooligosaccharides) reduced inflammation by mediating fatty acid oxidation via the Acly/Nrf2/NF-κB signaling pathway and inhibiting the differentiation of macrophages into pro-inflammatory M1 macrophages [211]. In addition to naturally occurring SDF, modified SDF have also shown potential in improving MASH.

Artichoke-derived SDFs processed by ultrafine grinding and microwave treatment exhibit more uniform particle sizes, which enhance their absorption and fermentability by gut microbiota. These modified fibers have been shown to hepatic steatosis and liver inflammation in MASH mouse models by inhibiting the TNF-α signaling pathway and suppressing IL-6 secretion. Notably, their hepatoprotective effects increase significantly in a dose-dependent manner [212]. Furthermore, SDF has been shown to improve inflammation and oxidative stress biomarkers in Zucker fatty rats, where fat accumulation, a risk factor for MASH, significantly increased the prevalence of MASH [213]. However, some studies have yielded contradictory results. In this respect, inulin partially restored the growth of bacteria impaired by a high-fat diet and regulated desaturase and elongase expression and activity. It also increased total unsaturated fatty acid levels [214]. Similarly, Komatsu et al. found that while inulin feeding improved liver inflammation, fibrosis, and elevated total lipoprotein and non-high-density lipoprotein cholesterol levels, it also raised triglyceride concentrations in the serum of rats [215]. Cordyceps polysaccharide, extracted from *Cordyceps sinensis*, prevented weight gain in MASH models but worsened liver fibrosis and steatosis [216]. This may be attributed to differences in SDF dosage and source. In the study by Komatsu et al., a high-inulin diet significantly increased serum triglyceride levels in mice, whereas a low-dose inulin intervention did not yield similar effects. Similarly, in the study by L. Chen et al., the authors employed *Cordyceps sinensis*-derived SDF (Cordyceps polysaccharide), which, despite demonstrating anti-inflammatory benefits in various inflammatory disease models, has been less extensively studied in the context of MASH. These findings suggests that the physiological impact of SDFs may vary depending on their molecular structure, origin, and disease context. It also highlights the necessity of disease-specific preclinical validation prior to clinical application. Comprehensive profiling of SDF composition and its interaction with host metabolism and gut microbiota is essential to avoid adverse outcomes and ensure therapeutic efficacy.

Beyond MASH, the role of SDF in alcoholic liver injury has also been explored. Co-administration of 20 % inulin and FOS was found to increase the abundance of *Bacteroides acidifaciens*. This bacterium regulates bile acid metabolism through bile salt hydrolase activity, leading to an increase in unconjugated bile acids, which in turn activate the intestinal FXR/FGF15 signaling pathway. This activation helps protect intestinal barrier function and promotes the expression of ornithine aminotransferase (OAT) in hepatocytes. OAT facilitates the conversion of ornithine, which accumulates in the liver during alcoholic injury, into glutamate, thereby providing substrates for hepatic detoxification and reducing hepatocellular damage caused by ammonia accumulation [217]. These findings suggest that while SDF generally shows beneficial effects in alleviating liver inflammatory responses, certain studies indicate that SDF may also exacerbate disease progression under specific conditions. In this respect, there is increasing evidence regarding the negative impact of SDF on liver cancer. For instance, inulin was found to induce jaundice-type hepatocellular carcinoma through bile acid accumulation, hepatocyte death, and neutrophil-driven liver inflammation, which could be mitigated by inhibiting fermentation, preventing bile acid buildup, and depleting fermentative bacteria [218,219]. Singh et al. found discovered that refined inulin promoted bile acid accumulation and liver cancer in a microbiome-dependent manner. This effect was counteracted by vancomycin, which selectively eliminated gut bacteria and reduced circulating secondary bile acids [220]. This divergence in outcomes may be attributed to the specific animal models used. For instance, the above studies employed Toll-like receptor 5-deficient mice as well as wild-type controls, suggesting that host genetic background and immune signaling pathways may significantly influence the outcomes of SDF interventions. TLR5-deficient mice are known to exhibit altered innate immune responses and microbial composition, potentially predisposing them to inflammation or metabolic disturbances under certain dietary conditions. Accordingly, these findings underscore the importance of selecting appropriate disease models when evaluating the safety and efficacy of SDFs and suggest that host–microbe interactions should be carefully considered in both preclinical and clinical trial design.

In conclusion, although numerous studies have demonstrated the therapeutic potential of SDF in treating liver inflammatory diseases and highlight its beneficial effects, there are a few studies suggesting that SDF may accelerate disease progression and promote liver cancer. This underscores the need for careful consideration when applying SDF-based treatments for liver conditions, particularly in cases with underlying cancer risk.

## Conclusion and perspective

SDF exerts significant anti-inflammatory effects in both gastrointestinal and distal organ inflammatory diseases. In the context of IBD, SDF modulates the gut microbiota to produce SCFAs, which subsequently regulate intestinal immune responses by upregulating anti-inflammatory cytokines (e.g., IL-10) and downregulating pro-inflammatory cytokines (e.g., TNF-α, IL-1β). Clinical and experimental evidence supports the potential of SDF, particularly inulin, pectin, β-glucan, and galacto-/fructo-oligosaccharides, as adjunctive treatments for IBD, IBS, and colorectal cancer, as well as distal inflammatory diseases such as neuroinflammation and MASH, via the gut–liver and gut–brain axes.

However, despite this promising therapeutic potential, several limitations currently hinder the broad clinical application of SDF. These include a lack of large-scale, multicenter clinical trials, an incomplete understanding of fiber-specific mechanisms of action, and uncertainties regarding optimal dosage, safety, and individualized response. Particularly in IBD, it remains unclear which types of SDF are most effective at different stages of disease progression. In addition, while SCFA-mediated benefits are widely acknowledged, the exact molecular pathways linking SDF fermentation to immune regulation in distal organs remain to be fully elucidated. Long-term safety assessments are also lacking, especially in patients with compromised gastrointestinal function.

To address these challenges, we propose several strategies for future clinical research. First, well-designed RCTs should include stratified patient selection (e.g., based on disease subtype, severity, microbiome composition, and fiber responsiveness), standardized dosing regimens for different SDF types, and multi-dimensional outcome metrics, including inflammatory markers, microbiota shifts, barrier integrity, and clinical remission indices. Second, dose-escalation and tolerability studies are necessary, especially in sensitive populations, such as patients with severe intestinal dysbiosis, impaired motility, or short bowel syndrome, who may be at risk of adverse gastrointestinal effects. Third, mechanistic investigations should be embedded within trials to explore host–microbiota–metabolite interactions and identify predictive biomarkers of response. Finally, personalized nutrition frameworks, supported by metagenomics and metabolomics profiling, may guide individualized SDF therapy and enhance therapeutic efficacy.

In summary, SDF holds considerable potential to become a valuable therapeutic adjunct in the management of IBD and other chronic inflammatory diseases. To fully realize this potential, future research should prioritize large-scale clinical validation, mechanistic clarification, patient stratification, and biotechnological innovation. Overcoming these challenges will be essential for translating the therapeutic promise of SDF into safe, targeted, and effective clinical interventions.

## Compliance with ethics requirements

This manuscript does not involve animal experiments

## CRediT authorship contribution statement

**Linkai Qu:** Investigation, Writing – original draft, Writing – review & editing, Project administration. **Ruining Zhang:** Methodology, Writing – review & editing. **Ziyu Chu:** Software, Writing – review & editing. **Jiapei Cai:** Validation, Visualization. **Yuhang He:** Validation, Visualization. **Xinyu Zhang:** Visualization. **Jiuxi Liu:** Visualization, Supervision. **Xufeng Xie:** Conceptualization, Writing – review & editing, Supervision, Project administration. **Yongguo Cao:** Conceptualization, Writing – review & editing, Supervision, Funding acquisition.

## Declaration of competing interest

The authors declare that they have no known competing financial interests or personal relationships that could have appeared to influence the work reported in this paper.

## References

[1] Hou K., Wu Z.-X., Chen X.-Y., Wang J.-Q., Zhang D., Xiao C. (2022). Microbiota in health and diseases. Sig Transduct Target Ther.

[2] Lu Y., Yuan X., Wang M., He Z., Li H., Wang J. (2022). Gut microbiota influence immunotherapy responses: mechanisms and therapeutic strategies. J Hematol Oncol.

[3] Arifuzzaman M., Collins N., Guo C.-J., Artis D. (2024). Nutritional regulation of microbiota-derived metabolites: Implications for immunity and inflammation. Immunity.

[4] Kim C.H. (2023). Complex regulatory effects of gut microbial short-chain fatty acids on immune tolerance and autoimmunity. Cell Mol Immunol.

[5] K M, Ec D, J W, F B. (2018). The impact of dietary fiber on gut microbiota in host health and disease. Cell Host Microbe.

[6] Clemente J.C., Manasson J., Scher J.U. (2018). The role of the gut microbiome in systemic inflammatory disease. BMJ.

[7] Hipsley E.H. (1953). Dietary “Fibre” and pregnancy toxaemia. BMJ.

[8] Anderson J.W., Midgley W.R., Wedman B. (1979). Fiber and diabetes. Diabetes Care.

[9] Vinik A.I., Jenkins D.J.A. (1988). Dietary fiber in management of diabetes. Diabetes Care.

[10] Bresalier R.S., Kim Y.S. (1985). Diet and colon cancer: putting the puzzle together. N Engl J Med.

[11] Liu K., Moss D., Persky V., Stamler J., Garside D., Soltero I. (1979). Dietary cholesterol, fat, and fibre, and colon-cancer mortality. Lancet.

[12] Zielinski G., DeVries J.W., Craig S.A., Bridges A.R. (2013). Dietary fiber methods in *Codex Alimentarius* : current status and ongoing discussions. Cereal Foods World.

[13] Food and Agriculture Organization/World Health Organization Codex Alimentarius Commission. Codex Alimentarius: Guidelines on Nutrition Labelling CAC/GL 2-1985. (FAO, 2010). n.d.

[14] Afshin A., Sur P.J., Fay K.A., Cornaby L., Ferrara G., Salama J.S. (2019). Health effects of dietary risks in 195 countries, 1990–2017: a systematic analysis for the Global Burden of Disease Study 2017. Lancet.

[15] Dietary Guidelines for Americans, 2020-2025 n.d.

[16] Chambers E.S., Byrne C.S., Frost G. (2019). Carbohydrate and human health: is it all about quality?. Lancet.

[17] Reynolds A., Mann J., Cummings J., Winter N., Mete E., Te Morenga L. (2019). Carbohydrate quality and human health: a series of systematic reviews and meta-analyses. Lancet.

[18] Stephen A.M., Champ M.-M.-J., Cloran S.J., Fleith M., Van Lieshout L., Mejborn H. (2017). Dietary fibre in Europe: current state of knowledge on definitions, sources, recommendations, intakes and relationships to health. Nutr Res Rev.

[19] European Commission (2017) Dietary Fibre. https://ec. europa.eu/jrc/en/health-knowledge-gateway/promotionprevention/nutrition/fibre n.d.

[20] Mayor S. Eating more fibre linked to reduced risk of non-communicable diseases and death, review finds. BMJ 2019:l159. 10.1136/bmj.l159.

[21] Sorboni S.G., Moghaddam H.S., Jafarzadeh-Esfehani R., Soleimanpour S. (2022). A comprehensive review on the role of the gut microbiome in human neurological disorders. Clin Microbiol Rev.

[22] Lee M., Chang E.B. (2021). Inflammatory bowel diseases (IBD) and the microbiome—Searching the crime scene for clues. Gastroenterology.

[23] Tursi A., Scarpignato C., Strate L.L., Lanas A., Kruis W., Lahat A. (2020). Colonic diverticular disease. Nat Rev Dis Primers.

[24] Ford A.C., Sperber A.D., Corsetti M., Camilleri M. (2020). Irritable bowel syndrome. Lancet.

[25] Ghavami A., Ziaei R., Talebi S., Barghchi H., Nattagh-Eshtivani E., Moradi S. (2023). Soluble fiber supplementation and serum lipid profile: a systematic review and dose-response meta-analysis of randomized controlled trials. Adv Nutr.

[26] Abdi R., Joye I.J. (2021). Prebiotic potential of cereal components. Foods.

[27] Thompson S.V., Hannon B.A., An R., Holscher H.D. (2017). Effects of isolated soluble fiber supplementation on body weight, glycemia, and insulinemia in adults with overweight and obesity: a systematic review and meta-analysis of randomized controlled trials. Am J Clin Nutr.

[28] Opperman C., Majzoobi M., Farahnaky A., Shah R., Van T.T.H., Ratanpaul V. (2025). Beyond soluble and insoluble: a comprehensive framework for classifying dietary fibre’s health effects. Food Res Int.

[29] Feng Y., Jin Q., Liu X., Lin T., Johnson A., Huang H. (2025). Advances in understanding dietary fiber: Classification, structural characterization, modification, and gut microbiome interactions. Comp Rev Food Sci Food Safe.

[30] Williams BA. “Dietary fibre”: moving beyond the “soluble/insoluble” classification for monogastric nutrition, with an emphasis on humans and pigs 2019. 10.1186/s40104-019-0350-9 PMC6537190 31149336

[31] Tanes C., Bittinger K., Gao Y., Friedman E.S., Nessel L., Roy Paladhi U. (2021). Role of dietary fiber in the recovery of the human gut microbiome and its metabolome. Cell Host Microbe.

[32] Verduci E., D’Elios S., Cerrato L., Comberiati P., Calvani M., Palazzo S. (2019). Cow’s milk substitutes for children: nutritional aspects of milk from different mammalian species, special formula and plant-based beverages. Nutrients.

[33] Jaye K., Li C.G., Chang D., Bhuyan D.J. (2022). The role of key gut microbial metabolites in the development and treatment of cancer. Gut Microbes.

[34] Takahashi T., Karita S., Ogawa N., Goto M. (2005). Crystalline cellulose reduces plasma glucose concentrations and stimulates water absorption by increasing the digesta viscosity in rats. J Nutr.

[35] Dhital S., Gidley M.J., Warren F.J. (2015). Inhibition of α-amylase activity by cellulose: Kinetic analysis and nutritional implications. Carbohydr Polym.

[36] Lovegrove A., Edwards C.H., De Noni I., Patel H., El S.N., Grassby T. (2017). Role of polysaccharides in food, digestion, and health. Crit Rev Food Sci Nutr.

[37] Ma X., Chen W., Yan T., Wang D., Hou F., Miao S. (2020). Comparison of citrus pectin and apple pectin in conjugation with soy protein isolate (SPI) under controlled dry-heating conditions. Food Chem.

[38] Liu Y., Lei F., He L., Xu W., Jiang J. (2020). Physicochemical characterization of galactomannans extracted from seeds of Gleditsia sinensis Lam and fenugreek. Comparison with commercial guar gum. Int J Biol Macromol.

[39] Borchani C., Fonteyn F., Jamin G., Destain J., Willems L., Paquot M. (2016). Structural characterization, technological functionality, and physiological aspects of fungal β-d-glucans: a review. Crit Rev Food Sci Nutr.

[40] Sikora P., Tosh S.M., Brummer Y., Olsson O. (2013). Identification of high β-glucan oat lines and localization and chemical characterization of their seed kernel β-glucans. Food Chem.

[41] Nasatto P., Pignon F., Silveira J., Duarte M., Noseda M., Rinaudo M. (2015). Methylcellulose, a cellulose derivative with original physical properties and extended applications. Polymers.

[42] Raninen K., Lappi J., Mykkänen H., Poutanen K. (2011). Dietary fiber type reflects physiological functionality: comparison of grain fiber, inulin, and polydextrose. Nutrit Rev.

[43] Fassarella M., Blaak E.E., Penders J., Nauta A., Smidt H., Zoetendal E.G. (2021). Gut microbiome stability and resilience: elucidating the response to perturbations in order to modulate gut health. Gut.

[44] Sommer F., Anderson J.M., Bharti R., Raes J., Rosenstiel P. (2017). The resilience of the intestinal microbiota influences health and disease. Nat Rev Microbiol.

[45] Tun H.M., Peng Y., Massimino L., Sin Z.Y., Parigi T.L., Facoetti A. (2024). Gut virome in inflammatory bowel disease and beyond. Gut.

[46] Hu Y., Chen Z., Xu C., Kan S., Chen D. (2022). Disturbances of the gut microbiota and microbiota-derived metabolites in inflammatory bowel disease. Nutrients.

[47] Shan Y., Lee M., Chang E.B. (2022). The gut microbiome and inflammatory bowel diseases. Annu Rev Med.

[48] Koh A., De Vadder F., Kovatcheva-Datchary P., Bäckhed F. (2016). From dietary fiber to host physiology: short-chain fatty acids as key bacterial metabolites. Cell.

[49] Sanders M.E., Merenstein D.J., Reid G., Gibson G.R., Rastall R.A. (2019). Probiotics and prebiotics in intestinal health and disease: from biology to the clinic. Nat Rev Gastroenterol Hepatol.

[50] Beukema M., Faas M.M., De Vos P. (2020). The effects of different dietary fiber pectin structures on the gastrointestinal immune barrier: impact via gut microbiota and direct effects on immune cells. Exp Mol Med.

[51] Stecher B., Hardt W.-D. (2011). Mechanisms controlling pathogen colonization of the gut. Curr Opin Microbiol.

[52] Xiong X., Tan B., Song M., Ji P., Kim K., Yin Y. (2019). Nutritional intervention for the Intestinal Development and Health of Weaned Pigs. Front Vet Sci.

[53] Lenders M., Brand E. (2022). Fabry disease – a multisystemic disease with gastrointestinal manifestations. Gut Microbes.

[54] Qu L., Li Y., Liu F., Fang Y., He J., Ma J. (2023). Microbiota-Gut-Brain Axis Dysregulation in Alzheimer’s Disease: Multi-Pathway Effects and Therapeutic potential. Aging Dis.

[55] Lu H., Xu X., Fu D., Gu Y., Fan R., Yi H. (2022). Butyrate-producing Eubacterium rectale suppresses lymphomagenesis by alleviating the TNF-induced TLR4/MyD88/NF-κB axis. Cell Host Microbe.

[56] Sharma A., Sharma G., Im S.-H. (2025). Gut microbiota in regulatory T cell generation and function: mechanisms and health implications. Gut Microbes.

[57] Guo X., Li J., Xu J., Zhang L., Huang C., Nie Y. (2025). Gut microbiota and epigenetic inheritance: implications for the development of IBD. Gut Microbes.

[58] Wu W., Sun M., Chen F., Cao A.T., Liu H., Zhao Y. (2017). Microbiota metabolite short-chain fatty acid acetate promotes intestinal IgA response to microbiota which is mediated by GPR43. Mucosal Immunol.

[59] Wu J., Yu G., Zhang X., Staiger M.P., Gupta T.B., Yao H. (2024). A fructan-type garlic polysaccharide upregulates immune responses in macrophage cells and in immunosuppressive mice. Carbohydr Polym.

[60] Cheng J., Ye K., Fu C., Zhou Y., Chen Y., Ma G. (2024). Comprehensive assessment of rice bran dietary fiber on gut microbiota composition and metabolism during in vitro fermentation. Food Res Int.

[61] Hezaveh K., Shinde R.S., Klötgen A., Halaby M.J., Lamorte S., Ciudad M.T. (2022). Tryptophan-derived microbial metabolites activate the aryl hydrocarbon receptor in tumor-associated macrophages to suppress anti-tumor immunity. Immunity.

[62] Ma Z., Akhtar M., Pan H., Liu Q., Chen Y., Zhou X. (2023). Fecal microbiota transplantation improves chicken growth performance by balancing jejunal Th17/Treg cells. Microbiome.

[63] Rosado-Rosa J.M., Parmar D., Rubakhin S.S., Shrout J.D., Sweedler J.V. (2025). D-Amino acids affect Pseudomonas aeruginosa biofilm and quorum sensing molecules in lung infection models developed under a cystic fibrosis environment. Sci Rep.

[64] Kepert I., Fonseca J., Müller C., Milger K., Hochwind K., Kostric M. (2017). D-tryptophan from probiotic bacteria influences the gut microbiome and allergic airway disease. J Allergy Clin Immunol.

[65] Murtas G., Sacchi S., Tedeschi G., Maffioli E., Notomista E., Cafaro V. (2021). Antimicrobial d-amino acid oxidase-derived peptides specify gut microbiota. Cell Mol Life Sci.

[66] Davis L.M.G., Martínez I., Walter J., Goin C., Hutkins R.W. (2011). Barcoded Pyrosequencing reveals that Consumption of Galactooligosaccharides results in a Highly specific Bifidogenic Response in Humans. PLoS One.

[67] Holscher H.D. (2017). Dietary fiber and prebiotics and the gastrointestinal microbiota. Gut Microbes.

[68] Vandeputte D., Falony G., Vieira-Silva S., Wang J., Sailer M., Theis S. (2017). Prebiotic inulin-type fructans induce specific changes in the human gut microbiota. Gut.

[69] Iovino P. (2014). Bloating and functional gastro-intestinal disorders: where are we and where are we going?. WJG.

[70] Friedman E.S., Bittinger K., Esipova T.V., Hou L., Chau L., Jiang J. (2018). Microbes vs. chemistry in the origin of the anaerobic gut lumen. PNAS.

[71] Yang K., Li G., Li Q., Wang W., Zhao X., Shao N. (2025). Distribution of gut microbiota across intestinal segments and their impact on human physiological and pathological processes. Cell Biosci.

[72] Litvak Y., Byndloss M.X., Bäumler A.J. (2018). Colonocyte metabolism shapes the gut microbiota. Science.

[73] Solanki S., Shah Y.M. (2024). Hypoxia-Induced Signaling in Gut and Liver Pathobiology. Annu Rev Pathol.

[74] Zheng L., Kelly C.J., Colgan S.P. (2015). Physiologic hypoxia and oxygen homeostasis in the healthy intestine. a Review in the Theme: Cellular responses to Hypoxia. Am J Phys Cell Phys.

[75] Lee J.-Y., Tsolis R.M., Bäumler A.J. (2022). The microbiome and gut homeostasis. Science.

[76] Kong C., Faas M.M., De Vos P., Akkerman R. (2020). Impact of dietary fibers in infant formulas on gut microbiota and the intestinal immune barrier. Food Funct.

[77] Chan S.Y., Choo W.S., Young D.J., Loh X.J. (2017). Pectin as a rheology modifier: Origin, structure, commercial production and rheology. Carbohydr Polym.

[78] Lin H., Han R., Wu W. (2024). Glucans and applications in drug delivery. Carbohydr Polym.

[79] Mensink M.A., Frijlink H.W., Van Der Voort M.K., Hinrichs W.L.J. (2015). Inulin, a flexible oligosaccharide I: Review of its physicochemical characteristics. Carbohydr Polym.

[80] Saeidy S., Petera B., Pierre G., Fenoradosoa T.A., Djomdi D., Michaud P. (2021). Plants arabinogalactans: from structures to physico-chemical and biological properties. Biotechnol Adv.

[81] Flores-Maltos D.A., Mussatto S.I., Contreras-Esquivel J.C., Rodríguez-Herrera R., Teixeira J.A., Aguilar C.N. (2016). Biotechnological production and application of fructooligosaccharides. Crit Rev Biotechnol.

[82] Manna S., Karmakar S., Sen O., Sinha P., Jana S., Jana S. (2024). Recent updates on guar gum derivatives in colon specific drug delivery. Carbohydr Polym.

[83] Mudgil D., Barak S. (2020). Mesquite gum (Prosopis gum): Structure, properties & applications - a review. Int J Biol Macromol.

[84] Ostrowski M. The Food Additive Xanthan Gum Drives Adaptation of the Human Gut Microbiota n.d. 10.1038/s41564-022-01093-0 PMC11537241 35365790

[85] Zheng J., Huang S., Zhao R., Wang N., Kan J., Zhang F. (2021). Effect of four viscous soluble dietary fibers on the physicochemical, structural properties, and in vitro digestibility of rice starch: a comparison study. Food Chem.

[86] Wang K., Duan F., Sun T., Zhang Y., Lu L. (2024). Galactooligosaccharides: Synthesis, metabolism, bioactivities and food applications. Crit Rev Food Sci Nutr.

[87] Li L., Zhu B., Yao Z., Jiang J. (2023). Directed preparation, structure–activity relationship and applications of alginate oligosaccharides with specific structures: a systematic review. Food Res Int.

[88] Liu F., Hou P., Zhang H., Tang Q., Xue C., Li R.W. (2021). Food‐grade carrageenans and their implications in health and disease. Comp Rev Food Sci Food Safe.

[89] Lu F. (2024). High-temperature glycosylation of saccharides to modify molecular conformation of egg white protein and its effect on the stability of high internal phase emulsions. Food Res Int.

[90] Walmagh M., Zhao R., Desmet T. (2015). Trehalose Analogues: latest Insights in Properties and Biocatalytic Production. IJMS.

[91] Chen Z, Mense AL, Brewer LR, Shi Y-C. Wheat bran arabinoxylans: Chemical structure, extraction, properties, health benefits, and uses in foods n.d. 10.1111/1541-4337.13366 38775125

[92] National Institute for Health and Care Excellence. Irritable bowel syndrome in adults: diagnosis and management (NICE, 2017). https://www.ncbi.nlm.nih.gov/books/NBK553734/ n.d. 32073807

[93] Lamb C.A., Kennedy N.A., Raine T., Hendy P.A., Smith P.J., Limdi J.K. (2019). British Society of Gastroenterology consensus guidelines on the management of inflammatory bowel disease in adults. Gut.

[94] World Gastroenterology Organisation WGO Practice Guideline – Diet and the Gut (WGO, 2018). n.d.

[95] Li M., Li S., Guo X., Guo C., Wang Y., Du Z. (2021). Discrete genetic loci in human gut Bacteroides thetaiotaomicron confer pectin metabolism. Carbohydr Polym.

[96] Wang W., Lin L., Zhao M. (2024). Simultaneously efficient dissolution and structural modification of chrysanthemum pectin: Targeting at proliferation of Bacteroides. Int J Biol Macromol.

[97] Yüksel E., Voragen A.G.J., Kort R. (2024). The pectin metabolizing capacity of the human gut microbiota. Crit Rev Food Sci Nutr.

[98] Zwolschen J.W., Vos A.P., Ariëns R.M.C., Schols H.A. (2024). In vitro batch fermentation of (un)saturated homogalacturonan oligosaccharides. Carbohydr Polym.

[99] Couto M.R., Gonçalves P., Magro F., Martel F. (2020). Microbiota-derived butyrate regulates intestinal inflammation: Focus on inflammatory bowel disease. Pharmacol Res.

[100] Han W., Wang N., Han M., Ban M., Sun T., Xu J. (2022). Reviewing the role of gut microbiota in the pathogenesis of depression and exploring new therapeutic options. Front Neurosci.

[101] Alonso-Allende J., Milagro F.I., Aranaz P. (2024). Health Effects and Mechanisms of Inulin Action in Human Metabolism. Nutrients.

[102] Tawfick M.M., Xie H., Zhao C., Shao P., Farag M.A. (2022). Inulin fructans in diet: Role in gut homeostasis, immunity, health outcomes and potential therapeutics. Int J Biol Macromol.

[103] Yang X., Zhang M., Liu Y., Wei F., Li X., Feng Y. (2023). Inulin-enriched Megamonas funiformis ameliorates metabolic dysfunction-associated fatty liver disease by producing propionic acid. npj Biofilms Microbiomes.

[104] Arpaia N., Campbell C., Fan X., Dikiy S., van der Veeken J., deRoos P. (2013). Metabolites produced by commensal bacteria promote peripheral regulatory T-cell generation. Nature.

[105] Ito T., Nakanishi Y., Shibata R., Sato N., Jinnohara T., Suzuki S. (2023). The propionate-GPR41 axis in infancy protects from subsequent bronchial asthma onset. Gut Microbes.

[106] Chambers E.S., Byrne C.S., Morrison D.J., Murphy K.G., Preston T., Tedford C. (2019). Dietary supplementation with inulin-propionate ester or inulin improves insulin sensitivity in adults with overweight and obesity with distinct effects on the gut microbiota, plasma metabolome and systemic inflammatory responses: a randomised cross-over trial. Gut.

[107] Yang Z., Su H., Lv Y., Tao H., Jiang Y., Ni Z. (2023). Inulin intervention attenuates hepatic steatosis in rats via modulating gut microbiota and maintaining intestinal barrier function. Food Res Int.

[108] Qiao H., Zhao T., Yin J., Zhang Y., Ran H., Chen S. (2022). Structural Characteristics of Inulin and Microcrystalline Cellulose and their effect on Ameliorating Colitis and Altering Colonic Microbiota in Dextran Sodium Sulfate-Induced Colitic mice. ACS Omega.

[109] Baldwin K.T., Carbajal K.S., Segal B.M., Giger R.J. (2015). Neuroinflammation triggered by β-glucan/dectin-1 signaling enables CNS axon regeneration. PNAS.

[110] Li X., Luo H., Ye Y., Chen X., Zou Y., Duan J. (2018). β‑glucan, a dectin‑1 ligand, promotes macrophage M1 polarization via NF‑κB/autophagy pathway. Int J Oncol.

[111] Uno A., Arima K., Shimazaki M., Ushida M., Amano K., Namikawa R. (2021). A novel β-glucan–oligonucleotide complex selectively delivers siRNA to APCs via Dectin-1. J Control Release.

[112] Li S., Peng H., Sun Y., Yang J., Wang J., Bai F. (2024). Yeast β-glucan attenuates dextran sulfate sodium-induced colitis: Involvement of gut microbiota and short-chain fatty acids. Int J Biol Macromol.

[113] Shi H., Yu Y., Lin D., Zheng P., Zhang P., Hu M. (2020). β-glucan attenuates cognitive impairment via the gut-brain axis in diet-induced obese mice. Microbiome.

[114] Thomas S.K., Wattenberg M.M., Choi-Bose S., Uhlik M., Harrison B., Coho H. (2023). Kupffer cells prevent pancreatic ductal adenocarcinoma metastasis to the liver in mice. Nat Commun.

[115] Zhong X., Wang G., Li F., Fang S., Zhou S., Ishiwata A. (2023). Immunomodulatory effect and Biological significance of β-Glucans. Pharmaceutics.

[116] Huang R., Zhang J., Sun M., Xu L., Kuang H., Xu C. (2025). Oat β-glucan enhances gut barrier function and maintains intestinal homeostasis in naturally aging mice. Int J Biol Macromol.

[117] Kocot A.M., Jarocka-Cyrta E., Drabińska N. (2022). Overview of the Importance of Biotics in Gut Barrier Integrity. IJMS.

[118] Caminero A., Guzman M., Libertucci J., Lomax A.E. (2023). The emerging roles of bacterial proteases in intestinal diseases. Gut Microbes.

[119] Oka P., Parr H., Barberio B., Black C.J., Savarino E.V., Ford A.C. (2020). Global prevalence of irritable bowel syndrome according to Rome III or IV criteria: a systematic review and meta-analysis. Lancet Gastroenterol Hepatol.

[120] Peery A.F., Crockett S.D., Murphy C.C., Lund J.L., Dellon E.S., Williams J.L. (2019). Burden and cost of gastrointestinal, liver, and pancreatic diseases in the United States: update 2018. Gastroenterology.

[121] Peery A.F., Crockett S.D., Murphy C.C., Jensen E.T., Kim H.P., Egberg M.D. (2022). Burden and cost of gastrointestinal, liver, and pancreatic diseases in the United States: update 2021. Gastroenterology.

[122] Sperber A.D., Bangdiwala S.I., Drossman D.A., Ghoshal U.C., Simren M., Tack J. (2021). Worldwide prevalence and burden of functional gastrointestinal disorders, results of rome foundation global study. Gastroenterology.

[123] Yuan Y., Wang X., Huang S., Wang H., Shen G. (2023). Low-level inflammation, immunity, and brain-gut axis in IBS: unraveling the complex relationships. Gut Microbes.

[124] Öhman L., Simrén M. (2010). Pathogenesis of IBS: role of inflammation, immunity and neuroimmune interactions. Nat Rev Gastroenterol Hepatol.

[125] Rao S.S.C., Yu S., Fedewa A. (2015). Systematic review: dietary fibre and FODMAP ‐restricted diet in the management of constipation and irritable bowel syndrome. Aliment Pharmacol Ther.

[126] Quigley, E. M. M. et al. Irritable bowel syndrome: a global perspective. World Gastroenterology Organisation Global Guidelines https:// www.worldgastroenterology.org/guidelines/ global-guidelines/irritable-bowel-syndrome-ibs/ irritable-bowel-syndrome-ibs-english (2015). n.d.

[127] Black C.J., Yuan Y., Selinger C.P., Camilleri M., Quigley E.M.M., Moayyedi P. (2020). Efficacy of soluble fibre, antispasmodic drugs, and gut–brain neuromodulators in irritable bowel syndrome: a systematic review and network meta-analysis. Lancet Gastroenterol Hepatol.

[128] Nagarajan N., Morden A., Bischof D., King E.A., Kosztowski M., Wick E.C. (2015). The role of fiber supplementation in the treatment of irritable bowel syndrome: a systematic review and meta-analysis. Eur J Gastroenterol Hepatol.

[129] Moayyedi P., Quigley E.M.M., Lacy B.E., Lembo A.J., Saito Y.A., Schiller L.R. (2014). The effect of Fiber Supplementation on Irritable Bowel Syndrome: a Systematic Review and Meta-analysis. Am J Gastroenterol.

[130] Lacy B.E., Pimentel M., Brenner D.M., Chey W.D., Keefer L.A., Long M.D. (2021). ACG clinical guideline: management of irritable bowel syndrome. Am J Gastroenterol.

[131] Ban Q.-Y., Liu M., Ding N., Chen Y., Lin Q., Zha J.-M. (2022). Nutraceuticals for the Treatment of IBD: current progress and future directions. Front Nutr.

[132] Ye Z., Wu Q., Yang S., Zhang Y., Zhou C., Liu M. (2023). Variety and quantity of dietary insoluble fiber intake from different sources and risk of new-onset hypertension. BMC Med.

[133] Yasukawa Z., Inoue R., Ozeki M., Okubo T., Takagi T., Honda A. (2019). Effect of repeated consumption of partially hydrolyzed guar gum on fecal characteristics and gut microbiota: a randomized, double-blind, placebo-controlled, and parallel-group clinical trial. Nutrients.

[134] Russo L., Andreozzi P., Zito F., Vozzella L., Savino I., Sarnelli G. (2015). Partially hydrolyzed guar gum in the treatment of irritable bowel syndrome with constipation: Effects of gender, age, and body mass index. Saudi J Gastroenterol.

[135] Kaplan G.G., Ng S.C. (2017). Understanding and preventing the global increase of inflammatory bowel disease. Gastroenterology.

[136] Ananthakrishnan A.N. (2015). Epidemiology and risk factors for IBD. Nat Rev Gastroenterol Hepatol.

[137] Kaplan G.G., Windsor J.W. (2021). The four epidemiological stages in the global evolution of inflammatory bowel disease. Nat Rev Gastroenterol Hepatol.

[138] Smith P.M., Howitt M.R., Panikov N., Michaud M., Gallini C.A., Bohlooly-Y M. (2013). The microbial metabolites, short-chain fatty acids, regulate colonic T _reg_ cell homeostasis. Science.

[139] Wedlake L., Slack N., Andreyev H.J.N., Whelan K. (2014). Fiber in the treatment and maintenance of inflammatory bowel disease: a systematic review of randomized controlled trials. Inflamm Bowel Dis.

[140] Zhang Z., Hyun J.E., Thiesen A., Park H., Hotte N., Watanabe H. (2020). Sex-specific differences in the gut microbiome in response to dietary fiber supplementation in IL-10-deficient mice. Nutrients.

[141] Lavelle A., Sokol H. (2020). Gut microbiota-derived metabolites as key actors in inflammatory bowel disease. Nat Rev Gastroenterol Hepatol.

[142] Cai J. (2021). Dioscin prevents DSS-induced colitis in mice with enhancing intestinal barrier function and reducing colon inflammation. Int Immunopharmacol.

[143] Liu J. (2023). Salidroside alleviates dextran sulfate sodium-induced colitis in mice by modulating the gut microbiota. Food Funct.

[144] Liu J., Cai J., Fan P., Dong X., Zhang N., Tai J. (2023). Salidroside protects mice from high-fat diet-induced obesity by modulating the gut microbiota. Int Immunopharmacol.

[145] Ni J., Wu G.D., Albenberg L., Tomov V.T. (2017). Gut microbiota and IBD: causation or correlation?. Nat Rev Gastroenterol Hepatol.

[146] James S.L., Christophersen C.T., Bird A.R., Conlon M.A., Rosella O., Gibson P.R. (2015). Abnormal fibre usage in UC in remission. Gut.

[147] Jakubczyk D., Leszczyńska K., Górska S. (2020). The effectiveness of probiotics in the treatment of inflammatory bowel disease (IBD)—A critical review. Nutrients.

[148] Deng M., Dan L., Ye S., Chen X., Fu T., Wang X. (2023). Higher dietary fibre intake is associated with lower risk of inflammatory bowel disease: prospective cohort study. Aliment Pharmacol Ther.

[149] Ananthakrishnan A.N., Khalili H., Konijeti G.G., Higuchi L.M., De Silva P., Korzenik J.R. (2013). A prospective study of long-term intake of dietary fiber and risk of Crohn’s disease and ulcerative colitis. Gastroenterology.

[150] Fernández-Bañares F., Hinojosa J., Sánchez-Lombraña J.L., Navarro E., Martínez-Salmerón J.F., García-Pugés A. (1999). Randomized clinical Trial of plantago ovata seeds (dietary fiber) as compared with mesalamine in maintaining remission in ulcerative colitis. Am J Gastroenterol.

[151] Hallert C., Kaldma M., Petersson B.G. (1991). Ispaghula Husk May Relieve Gastrointestinal Symptoms in Ulcerative Colitis in Remission. Scand J Gastroenterol.

[152] Faghfoori Z., Navai L., Shakerhosseini R., Somi M.H., Nikniaz Z., Norouzi M.F. (2011). Effects of an oral supplementation of germinated barley foodstuff on serum tumour necrosis factor-α, interleukin-6 and -8 in patients with ulcerative colitis. Ann Clin Biochem.

[153] Steed H., Macfarlane G.T., Blackett K.L., Bahrami B., Reynolds N., Walsh S.V. (2010). Clinical trial: the microbiological and immunological effects of synbiotic consumption – a randomized double‐blind placebo‐controlled study in active Crohn’s disease. Aliment Pharmacol Ther.

[154] Jones V.A., Workman E., Freeman A.H., Dickinson R.J., Wilson A.J., Hunter J.O. (1985). Crohn’s disease: maintenance of remission by diet. Lancet.

[155] Pujo J., Petitfils C., Le Faouder P., Eeckhaut V., Payros G., Maurel S. (2021). Bacteria-derived long chain fatty acid exhibits anti-inflammatory properties in colitis. Gut.

[156] Cui L., Zeng H., Hou M., Li Z., Mu C., Zhu W. (2023). Lactiplantibacillus plantarum L47 and inulin alleviate enterotoxigenic Escherichia coli induced ileal inflammation in piglets by upregulating the levels of α-linolenic acid and 12,13-epoxyoctadecenoic acid. Anim Nutr.

[157] Wu T., Wang G., Xiong Z., Xia Y., Song X., Zhang H. (2022). Probiotics Interact with Lipids Metabolism and Affect Gut Health. Front Nutr.

[158] Su Y., Wang W., Meng X. (2022). Revealing the Roles of MOAP1 in Diseases. A Review.

[159] Siegel R.L., Wagle N.S., Cercek A., Smith R.A., Jemal A. (2023). Colorectal cancer statistics, 2023. CA A Cancer J Clinicians.

[160] Chan C.W., Lee P.H. (2016). Association between dietary fibre intake with cancer and all‐cause mortality among 15 740 adults: the N ational H ealth and N utrition E xamination S urvey III. J Human Nutrition Diet.

[161] Arayici M.E., Mert-Ozupek N., Yalcin F., Basbinar Y., Ellidokuz H. (2022). Soluble and Insoluble Dietary Fiber Consumption and Colorectal Cancer Risk: a Systematic Review and Meta-Analysis. Nutr Cancer.

[162] Zhang S.-L., Mao Y.-Q., Zhang Z.-Y., Li Z.-M., Kong C.-Y., Chen H.-L. (2021). Pectin supplement significantly enhanced the anti-PD-1 efficacy in tumor-bearing mice humanized with gut microbiota from patients with colorectal cancer. Theranostics.

[163] Prado S.B.R.D., Santos G.R.C., Mourão P.A.S., Fabi J.P. (2019). Chelate-soluble pectin fraction from papaya pulp interacts with galectin-3 and inhibits colon cancer cell proliferation. Int J Biol Macromol.

[164] Zhao W., Ren A., Shan S., Li Z., Su R., Yang R. (2024). Inhibitory Effects of Soluble Dietary Fiber from Foxtail Millet on Colorectal Cancer by the Restoration of Gut Microbiota. J Agric Food Chem.

[165] Kopiasz Ł., Dziendzikowska K., Oczkowski M., Harasym J., Gromadzka-Ostrowska J. (2024). Low-molar-mass oat beta-glucan impacts autophagy and apoptosis in early stages of induced colorectal carcinogenesis in rats. Int J Biol Macromol.

[166] Wang Q., Wang P., Xiao Z. (2018). Resistant starch prevents tumorigenesis of dimethylhydrazine-induced colon tumors via regulation of an ER stress-mediated mitochondrial apoptosis pathway. Int J Mol Med.

[167] Nangia-Makker P., Hogan V., Honjo Y., Baccarini S., Tait L., Bresalier R. (2002). Inhibition of human cancer cell growth and metastasis in nude mice by oral intake of modified citrus pectin. JNCI.

[168] Corrêa R.O., Castro P.R., Fachi J.L., Nirello V.D., El-Sahhar S., Imada S. (2023). Inulin diet uncovers complex diet-microbiota-immune cell interactions remodeling the gut epithelium. Microbiome.

[169] Yang J., Wei H., Lin Y., Chu E.S.H., Zhou Y., Gou H. (2024). High soluble fiber promotes colorectal tumorigenesis through modulating gut microbiota and metabolites in mice. Gastroenterology.

[170] Okazaki H, Nishimune T, Matsuzaki H, Miura T, Morita S, Yanagimoto Y, et al. Increased incidence rate of colorectal tumors due to the intake of a soluble dietary fiber in rat chemical carcinogenesis can be suppressed by substituting partially an insoluble dietary fiber for the soluble one n.d. 10.1002/ijc.10493 12115518

[171] Poh L. The role of inflammasomes in vascular cognitive impairment 2022. 10.1186/s13024-021-00506-8 PMC8744307 35000611

[172] Bradburn S., Murgatroyd C., Ray N. (2019). Neuroinflammation in mild cognitive impairment and Alzheimer’s disease: a meta-analysis. Ageing Res Rev.

[173] Alexander C., Swanson K.S., Fahey G.C., Garleb K.A. (2019). Perspective: physiologic importance of short-chain fatty acids from nondigestible carbohydrate fermentation. Adv Nutr.

[174] Mitusova K. Overcoming the blood–brain barrier for the therapy of malignant brain tumor: current status and prospects of drug delivery approaches 2022. 10.1186/s12951-022-01610-7 PMC9479308 36109754

[175] Chandra S., Sisodia S.S., Vassar R.J. (2023). The gut microbiome in Alzheimer’s disease: what we know and what remains to be explored. Mol Neurodegen.

[176] Matt S.M., Allen J.M., Lawson M.A., Mailing L.J., Woods J.A., Johnson R.W. (2018). Butyrate and dietary soluble fiber improve neuroinflammation associated with aging in mice. Front Immunol.

[177] Vailati-Riboni M., Rund L., Caetano-Silva M.E., Hutchinson N.T., Wang S.S., Soto-Díaz K. (2022). Dietary fiber as a counterbalance to age-related microglial cell dysfunction. Front Nutr.

[178] Caetano-Silva M.E., Rund L., Hutchinson N.T., Woods J.A., Steelman A.J., Johnson R.W. (2023). Inhibition of inflammatory microglia by dietary fiber and short-chain fatty acids. Sci Rep.

[179] Li X.-W., Qiu F., Liu Y., Chen L.-J., Li J.-H., Liu J.-L. (2023). Inulin alleviates neuroinflammation and oxidative stress induced by perinatal 2-ethylhexyl diphenyl phosphate (EHDPHP) exposure in female mice and offspring. Ecotoxicol Environ Saf.

[180] Zou H., Gao H., Liu Y., Zhang Z., Zhao J., Wang W. (2024). Dietary inulin alleviated constipation induced depression and anxiety-like behaviors: Involvement of gut microbiota and microbial metabolite short-chain fatty acid. Int J Biol Macromol.

[181] Hoffman J.D., Yanckello L.M., Chlipala G., Hammond T.C., McCulloch S.D., Parikh I. (2019). Dietary inulin alters the gut microbiome, enhances systemic metabolism and reduces neuroinflammation in an APOE4 mouse model. PLoS One.

[182] Liu Z., Li L., Ma S., Ye J., Zhang H., Li Y. (2020). High-Dietary Fiber Intake Alleviates Antenatal Obesity-Induced Postpartum Depression: Roles of Gut Microbiota and Microbial Metabolite Short-chain Fatty Acid involved. J Agric Food Chem.

[183] Wang L., Wang Z., Lan Y., Tuo Y., Ma S., Liu X. (2023). Inulin Attenuates Blood–Brain Barrier Permeability and Alleviates Behavioral Disorders by Modulating the TLR4/MyD88/NF-κB Pathway in mice with Chronic stress. J Agric Food Chem.

[184] Petrovsky N., Larena M., Siddharthan V., Prow N.A., Hall R.A., Lobigs M. (2013). An Inactivated Cell Culture Japanese Encephalitis Vaccine (JE-ADVAX) Formulated with Delta Inulin Adjuvant Provides Robust Heterologous Protection against West Nile Encephalitis via Cross-protective memory B Cells and Neutralizing Antibody. J Virol.

[185] Bielefeldt-Ohmann H., Prow N.A., Wang W., Tan C.S., Coyle M., Douma A. (2014). Safety and immunogenicity of a delta inulin-adjuvanted inactivated Japanese encephalitis virus vaccine in pregnant mares and foals. Vet Res.

[186] Lobigs M., Pavy M., Hall R.A., Lobigs P., Cooper P., Komiya T. (2010). An inactivated Vero cell-grown Japanese encephalitis vaccine formulated with Advax, a novel inulin-based adjuvant, induces protective neutralizing antibody against homologous and heterologous flaviviruses. J Gen Virol.

[187] Cui Z., Gong Y., Luo X., Zheng N., Tan S., Liu S. (2023). β-Glucan alleviates goal-directed behavioral deficits in mice infected with Toxoplasma gondii. Parasites Vectors.

[188] Heng Y., Zhang X., Borggrewe M., Van Weering H.R.J., Brummer M.L., Nijboer T.W. (2021). Systemic administration of β-glucan induces immune training in microglia. J Neuroinflammation.

[189] Pan W., Jiang P., Zhao J., Shi H., Zhang P., Yang X. (2021). β-Glucan from Lentinula edodes prevents cognitive impairments in high-fat diet-induced obese mice: involvement of colon-brain axis. J Transl Med.

[190] Zhang Q., Zhao W., Hou Y., Song X., Yu H., Tan J. (2023). β‐Glucan attenuates cognitive impairment of APP / PS1 mice via regulating intestinal flora and its metabolites. CNS Neurosci Ther.

[191] Xu M., Mo X., Huang H., Chen X., Liu H., Peng Z. (2020). Yeast β-glucan alleviates cognitive deficit by regulating gut microbiota and metabolites in Aβ1–42-induced AD-like mice. Int J Biol Macromol.

[192] Lacasa M., Alegre-Martin J., Sentañes R.S., Varela-Sende L., Jurek J., Castro-Marrero J. (2023). Yeast Beta-Glucan Supplementation with Multivitamins Attenuates Cognitive Impairments in individuals with Myalgic Encephalomyelitis/Chronic Fatigue Syndrome: a Randomized, Double-blind. Placebo-Controll Trial Nutr.

[193] Berer K., Martínez I., Walker A., Kunkel B., Schmitt-Kopplin P., Walter J. (2018). Dietary non-fermentable fiber prevents autoimmune neurological disease by changing gut metabolic and immune status. Sci Rep.

[194] Church J.S., Bannish J.A.M., Adrian L.A., Rojas Martinez K., Henshaw A., Schwartzer J.J. (2023). Serum short chain fatty acids mediate hippocampal BDNF and correlate with decreasing neuroinflammation following high pectin fiber diet in mice. Front Neurosci.

[195] Paiva I.H.R.D., Maciel L.M., Silva R.S.D., Mendonça I.P., Souza J.R.B.D., Peixoto C.A. (2024). Prebiotics modulate the microbiota–gut–brain axis and ameliorate anxiety and depression-like behavior in HFD-fed mice. Food Res Int.

[196] Savignac H.M., Couch Y., Stratford M., Bannerman D.M., Tzortzis G., Anthony D.C. (2016). Prebiotic administration normalizes lipopolysaccharide (LPS)-induced anxiety and cortical 5-HT2A receptor and IL1-β levels in male mice. Brain Behav Immun.

[197] Zhang S., Lv S., Li Y., Wei D., Zhou X., Niu X. (2023). Prebiotics modulate the microbiota–gut–brain axis and ameliorate cognitive impairment in APP/PS1 mice. Eur J Nutr.

[198] Lee Y.-S., Lai D.-M., Huang H.-J., Lee-Chen G.-J., Chang C.-H., Hsieh-Li H.M. (2021). Prebiotic Lactulose Ameliorates the Cognitive Deficit in Alzheimer’s Disease Mouse Model through Macroautophagy and Chaperone-Mediated Autophagy Pathways. J Agric Food Chem.

[199] Yang X.-D., Wang L.-K., Wu H.-Y., Jiao L. (2018). Effects of prebiotic galacto-oligosaccharide on postoperative cognitive dysfunction and neuroinflammation through targeting of the gut-brain axis. BMC Anesthesiol.

[200] De Paiva I.H.R., Da Silva R.S., Mendonça I.P., Duarte-Silva E., Botelho De Souza J.R., Peixoto C.A. (2023). Fructooligosaccharide (FOS) and Galactooligosaccharide (GOS) Improve Neuroinflammation and Cognition by Up-regulating IRS/PI3K/AKT Signaling Pathway in Diet-induced Obese mice. J Neuroimmune Pharmacol.

[201] Liu Q., Xi Y., Wang Q., Liu J., Li P., Meng X. (2021). Mannan oligosaccharide attenuates cognitive and behavioral disorders in the 5xFAD Alzheimer’s disease mouse model via regulating the gut microbiota-brain axis. Brain Behav Immun.

[202] Yin Q., Chen J., Ma S., Dong C., Zhang Y., Hou X. (2020). Pharmacological inhibition of galectin-3 ameliorates diabetes-associated cognitive impairment, oxidative stress and neuroinflammation in vivo and in vitro. JIR.

[203] Scordino M., Urone G., Frinchi M., Valenza C., Bonura A., Cipollina C. (2024). Anti-apoptotic and anti-inflammatory properties of grapefruit integropectin on human microglial HMC3 cell line. Cells.

[204] Wang Y., Hou J.-L. (2015). Current strategies for quantitating fibrosis in liver biopsy. Chin Med J (Engl).

[205] Huang DQ, El-Serag HB, Loomba R. Global epidemiology of NAFLD-related HCC: trends, predictions, risk factors and prevention, 2022. 10.1038/s41575-020-00381-6 PMC8016738 33349658

[206] Friedman S.L., Neuschwander-Tetri B.A., Rinella M., Sanyal A.J. (2018). Mechanisms of NAFLD development and therapeutic strategies. Nat Med.

[207] Wei W., Wong C.C., Jia Z., Liu W., Liu C., Ji F. (2023). Parabacteroides distasonis uses dietary inulin to suppress NASH via its metabolite pentadecanoic acid. Nat Microbiol.

[208] Dong Y., Guo Y., Li Q., Zhao Y., Cao J. (2024). Soluble dietary fiber from Dendrocalamus brandisii (Munro) Kurz shoot improves liver injury by regulating gut microbial disorder in mice. Food Chem: X.

[209] Kawauchi S., Horibe S., Sasaki N., Tanahashi T., Mizuno S., Hamaguchi T. (2019). Inhibitory Effects of Sodium Alginate on Hepatic Steatosis in mice Induced by a Methionine- and Choline-Deficient Diet. Mar Drugs.

[210] Wang P., Sun J., Zhao W., Wang D., Ma Y., Zhao Y. (2024). Tomato pectin ameliorated hepatic steatosis in high-fat-diet mice by modulating gut microbiota and bile acid metabolism. J Agric Food Chem.

[211] Shao J., Ge T., Wei Y., Zhou Y., Shi M., Liu H. (2022). Co-interventions with *Clostridium butyricum* and soluble dietary fiber targeting the gut microbiota improve MAFLD *via* the Acly/Nrf2/NF-κB signaling pathway. Food Funct.

[212] Wang Y., He B., Zhang L., Zhu R., Huang L. (2023). Physicochemical properties of superfine grinding-microwave modified artichoke soluble dietary fiber and their alleviation of alcoholic fatty liver in mice. Front Nutr.

[213] Sánchez D., Quiñones M., Moulay L., Muguerza B., Miguel M., Aleixandre A. (2011). Soluble fiber-enriched diets improve inflammation and oxidative stress biomarkers in Zucker fatty rats. Pharmacol Res.

[214] Albouery M., Bretin A., Buteau B., Grégoire S., Martine L., Gambert S. (2021). Soluble Fiber Inulin Consumption Limits Alterations of the Gut Microbiota and Hepatic Fatty Acid Metabolism Caused by High-Fat Diet. Nutrients.

[215] Komatsu Y., Aoyama K., Yoneda M., Ashikawa S., Nakano S., Kawai Y. (2021). The prebiotic fiber inulin ameliorates cardiac, adipose tissue, and hepatic pathology, but exacerbates hypertriglyceridemia in rats with metabolic syndrome. Am J Physiol-Heart Circul Physiol.

[216] Chen L., Zhang L., Wang W., Qiu W., Liu L., Ning A. (2020). Polysaccharides isolated from Cordyceps Sinensis contribute to the progression of NASH by modifying the gut microbiota in mice fed a high-fat diet. PLoS One.

[217] Shen H., Zhou L., Zhang H., Yang Y., Jiang L., Wu D. (2024). Dietary fiber alleviates alcoholic liver injury via Bacteroides acidifaciens and subsequent ammonia detoxification. Cell Host Microbe.

[218] Singh V., Yeoh B.S., Chassaing B., Xiao X., Saha P., Aguilera Olvera R. (2018). Dysregulated microbial fermentation of soluble fiber induces cholestatic liver cancer. Cell.

[219] Gallage S., Kotsiliti E., Heikenwalder M. (2018). When soluble fibers meet hepatocellular carcinoma: the dark side of fermentation. Cell Metab.

[220] Singh V., Yeoh B.S., Abokor A.A., Golonka R.M., Tian Y., Patterson A.D. (2020). Vancomycin prevents fermentable fiber-induced liver cancer in mice with dysbiotic gut microbiota. Gut Microbes.

